# Molecular basis for metabolite channeling in a ring opening enzyme of the phenylacetate degradation pathway

**DOI:** 10.1038/s41467-019-11931-1

**Published:** 2019-09-11

**Authors:** Nitish Sathyanarayanan, Giuseppe Cannone, Lokesh Gakhar, Nainesh Katagihallimath, Ramanathan Sowdhamini, Subramanian Ramaswamy, Kutti R. Vinothkumar

**Affiliations:** 1 Institute for Stem Cell Science and Regenerative Medicine, GKVK Campus, Bellary Road, Bangalore, India; 2 Institute of Trans-Disciplinary Health Sciences and Technology (TDU), Bangalore, India; 3 Medical Research Council Laboratory of Molecular Biology, Francis Crick Avenue, Cambridge, UK; 4 Protein Crystallography Facility and Department of Biochemistry, Carver College of Medicine, University of Iowa, Iowa City, IA USA; 5 National Centre for Biological Sciences TIFR, GKVK Campus, Bellary Road, Bangalore, India; 6 Present Address: Bugworks Research India Pvt. Ltd., Centre for Cellular and Molecular Platforms, National Centre for Biological Sciences TIFR, GKVK Campus, Bellary Road, Bangalore, India

**Keywords:** Multienzyme complexes, Bacterial structural biology, Cryoelectron microscopy

## Abstract

Substrate channeling is a mechanism for the internal transfer of hydrophobic, unstable or toxic intermediates from the active site of one enzyme to another. Such transfer has previously been described to be mediated by a hydrophobic tunnel, the use of electrostatic highways or pivoting and by conformational changes. The enzyme PaaZ is used by many bacteria to degrade environmental pollutants. PaaZ is a bifunctional enzyme that catalyzes the ring opening of oxepin-CoA and converts it to 3-oxo-5,6-dehydrosuberyl-CoA. Here we report the structures of PaaZ determined by electron cryomicroscopy with and without bound ligands. The structures reveal that three domain-swapped dimers of the enzyme form a trilobed structure. A combination of small-angle X-ray scattering (SAXS), computational studies, mutagenesis and microbial growth experiments suggests that the key intermediate is transferred from one active site to the other by a mechanism of electrostatic pivoting of the CoA moiety, mediated by a set of conserved positively charged residues.

## Abstract (web summary)

The bacterial enzyme PaaZ is involved in the breakdown of environmental pollutants via the aerobic-anaerobic hybrid pathway but its substrate transfer mechanism is not fully understood. Here, the authors present cryoEM structures of free and ligand-bound PaaZ that suggest a mechanism for internal substrate channeling.

## Introduction

Substrate channeling is the direct transfer of an intermediate between the catalytic sites of a two-step reaction without its release to the bulk solvent. The two catalytic sites can either be present on different domains within a multi-domain enzyme or on different proteins which assemble to form a complex^1^. The efficient transfer of intermediates can be achieved through formation of molecular tunnels^2,3^ or through electrostatic residues mediating transfer^4^. In the case of the bacterial fatty acid β-oxidation multi-enzyme complex, Ishikawa et al., described a mechanism where Coenzyme A (CoA) binding to a single site acts as a pivot to transfer the intermediate from 2-enoyl-CoA hydratase (ECH) to L-3-hydroxyacyl-CoA dehydrogenase (HACD) active sites, while the transfer to the third component 3-ketoacyl-CoA thiolase (KACT) is proposed to occur through a large conformational change^5^. The mechanism of substrate channeling is argued to have entropic advantage, as it prevents the loss of intermediates into bulk solvent or to other competing metabolic pathways. The mechanism also prevents toxic intermediates from diffusing into the solvent and the formation of dead end products^6^.

Aromatic compounds represent a major class of environmental pollutants. The availability of oxygen primarily determines the mechanism by which the dearomatization of the ring occurs^7^. Under aerobic conditions, oxygen is utilized by monooxygenase or dioxygenase systems for both hydroxylation and subsequent cleavage of the ring^8,9^. Under anaerobic conditions, the substrate is first activated by covalent linkage to CoA to form a CoA-thioester bond. In the next step, energy from ATP hydrolysis or electrons from flavin is used for ring reduction^10,11^. There are two major challenges in ring opening of these compounds. The first is to break open the aromatic ring that is stabilized by resonance energy. The second is to prevent the formation of dead end products or unstable intermediates that can rearomatize^12,13^.

**Fig. 1:**
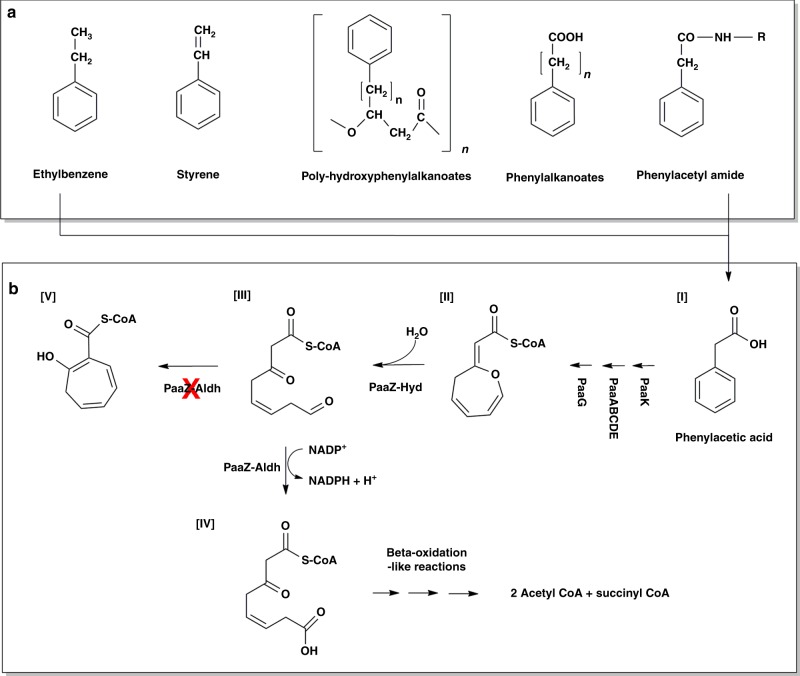
Overview of phenyl acetic acid (paa) degradation pathway. **a** Several environmental pollutants such as styrene, ethylbenzene and others converge to paa through peripheral pathways. **b** The first reaction in paa pathway is catalyzed by PaaK, which covalently links paa (I) with CoA giving rise to paa-CoA. The next reaction is catalyzed by a multi-component monooxygenase system PaaABCDE followed by isomerase PaaG to yield Oxepin CoA (II), which is the substrate for PaaZ. PaaZ-Hyd performs the hydratase reaction to yield 3-oxo-5,6-dehydrosuberyl-CoA semialdehyde (III). PaaZ-Aldh domain reduces the semi-aldehyde intermediate to a ring opened 3-oxo-5,6-dehydrosuberyl-CoA (IV) which is further reduced to acetyl-CoA and succinyl-CoA via a reaction similar to beta-oxidation. If either PaaZ-Aldh is impaired or if the enzyme is not supplemented with NADP^+^, the semialdehyde intermediate (III) rapidly forms a dead end product 2-hydroxycyclohepta-1,4,6-triene-1-carboxyl-CoA (V), which cannot be rescued by the addition of functional aldehyde dehydrogenase domain. Figure based on Fuchs et al.^15^

In conditions where the availability of oxygen is limited, microorganisms have evolved mechanisms to breakdown the aromatic ring by combining features of both aerobic and anaerobic pathways^14^. One such mechanism was observed in the degradation of phenyl acetic acid (paa). Several environmental pollutants such as styrene, trans-styrylacetic acid, 2-phenylethylamine, phenylalkanoic acids converge to paa through peripheral pathways (Fig. 1a)^15^. The phenylacetic acid degradation pathway found in several bacteria^16,17^ is referred to as a ‘hybrid pathway’ because it incorporates features of both the aerobic and anaerobic mechanisms of ring cleavage (Fig. 1b). The crucial ring opening reaction (Fig. 1b II,III,IV) is catalyzed by PaaZ, which contains an N-terminal aldehyde dehydrogenase domain and a C-terminal enoyl-CoA hydratase domain^18^. Using kinetic experiments, Teufel et. al., have previously shown that a “dead end” product (Fig. 1b V) is obtained either when the catalytic residue of aldehyde dehydrogenase domain is mutated to render the enzyme non-functional or if the reaction is not supplemented with NADP^+^. Addition of a functional aldehyde dehydrogenase domain in trans did not result in the rescue of the reaction suggesting that the intermediate (Fig. 1b III) must be channeled from the hydratase domain to the dehydrogenase domain^16^.

A comprehensive biochemical characterization of the entire paa pathway including PaaZ was first reported by Teufel et al.^19^. The structural characterization of several enzymes in the paa pathway has been reported earlier^20–22^. However, the structure of PaaZ, the enzyme mediating ring cleavage, has remained elusive. The individual domains of PaaZ show high sequence similarity to dehydrogenase and hydratase domains whose structures are known. This suggests that the folds of the individual domains of PaaZ are likely to be similar to the known structures^23,24^. However, the quarternary structure of PaaZ, the relative arrangement of the two domains with different enzymatic functions and the manner in which the substrate is transferred from one domain to the other remained unknown.

Here, using single-particle electron cryomicroscopy (cryoEM) we report the structures of PaaZ with and without bound ligands. The structures and the relative position of the ligand in both the hydratase and dehydrogenase domains provide a plausible hypothesis on the substrate transfer mechanism.

## Results

### The electron cryomicroscopy structure of PaaZ from *E. coli*

Our efforts to obtain structure of PaaZ with X-ray crystallography did not yield diffracting crystals. Several attempts were made to improve the quality of the crystals including methods like post crystallization treatments^25^, lysine modifications^26,27^ limited proteolysis, etc., but were not successful. Then, we pursued structure determination by single particle cryoEM. Initially, cryoEM grids of PaaZ were made on holey-carbon grids on ice without any support material (micrograph shown in Supplementary Fig. 1a). Visual inspection of PaaZ images clearly showed species that were trilobal in shape (top and bottom view—marked in red in Supplementary Fig. 1a, b) and elongated structures (side views—marked in green in Supplementary Fig. 1b). Apart from the trilobed structures, occasional dissociated dimers were also observed in the micrographs (marked in blue in Supplementary Fig. 1a). This could be a result of dissociation occurring during blotting and freezing. Data collected from images on ice resulted in a reconstruction to about 4 Å resolution, revealing the secondary structural features and a number of side chain densities. The protein on ice did not yield an even spread of molecules and had a tendency to clump, inhibiting high-resolution data collection (Supplementary Fig. 1a). Using graphene oxide on Ultrafoil gold grids and low protein concentration (the peak fraction from gel-filtration used for dilution) yielded a particle distribution that was used to collect higher resolution data with a Falcon 3 detector in counting mode (Supplementary Fig. 1b). A representative 2D class average is shown in Supplementary Fig. 1c and these reference-free class averages were then used to generate an initial model. It is important to note that the search space to find the right combination to improve the behavior of proteins on cryoEM grids is wide. There are perhaps conditions that have not been tested that will provide an even better distribution of the PaaZ on cryoEM grids.

### Architecture of PaaZ

**Fig. 2:**
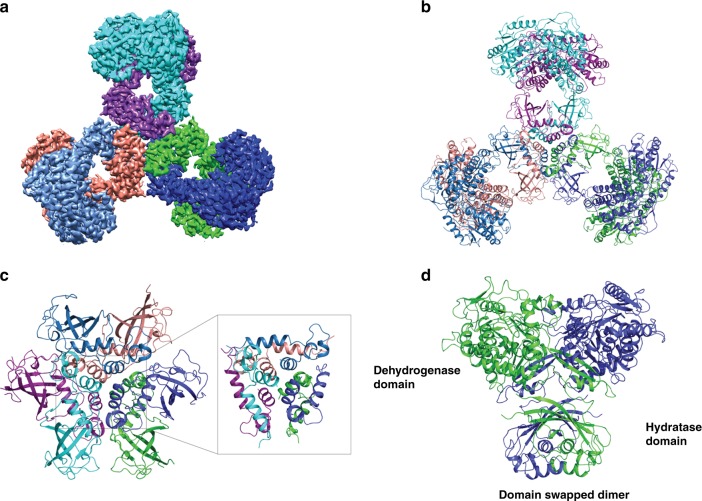
Architecture of PaaZ. **a** CryoEM map of substrate-free PaaZ revealing a trilobed architecture. The density corresponding to each monomer is colored individually, clearly showing the domain swap in each module. **b** The model derived from the cryoEM map with each polypeptide colored as in panel **a**. The final model consists of residues 2–679 with only the loop regions defined poorly. **c** Six monomers of the hydratase domain form the central inner core. Short helices from each monomer form two layers with an additional helix that lies perpendicular and wraps around to form a tight knit core (inset). **d** Two polypeptides of PaaZ (green and blue) form a module with the domain of the C-terminal hydratase, which is swapped between monomers

**Fig. 3:**
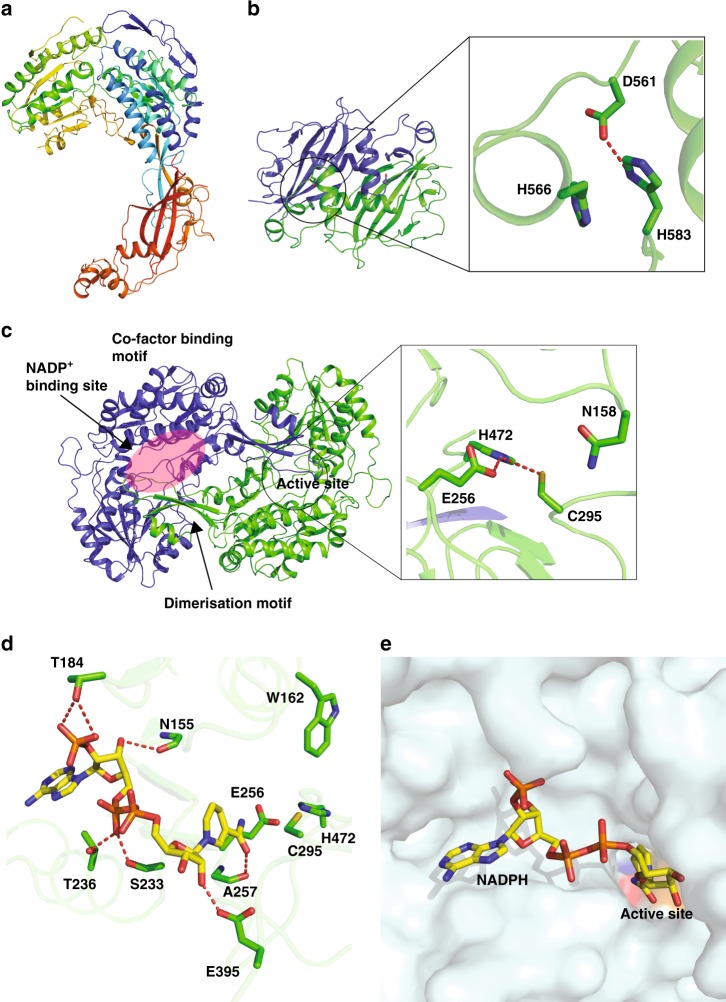
Enzymatic domains in PaaZ and the cofactor binding site. **a** The polypeptide of the PaaZ monomer color coded in rainbow with the N-terminus in blue and C-terminus in red. **b** The hydratase domain is made of five β-sheets with three additional helices and the monomers are colored in blue and green. The active site comprising D561 and H566 of one monomer is closer to the substrate-binding tunnel of the other monomer, and a hydrogen bond (3.3 Å) is observed between H583 and D561. **c** The dehydrogenase domain can be divided into three sub-domains: co-factor binding, catalytic and dimerization^23^. They are made of a mixture of α-helices and β-strands. The active site of the dehydrogenase domain consists of nucleophile cysteine C295 and E256. A histidine residue (H472) is in hydrogen bonding distance to E256 and ~3.55 Å to C295. The distance between E256 and C295 is 3.5 Å. The monomers of the dehydrogenase domain are colored in blue and green. **d** The interactions of NADPH observed in the structure. Numerous interactions with polar and charged residues of the enzyme stabilize the NADPH binding. **e** The NADPH binding groove and the active site residues are colored (C295 in orange, E256 in red and H472 in blue). The nicotinamide moiety points towards the active site cavity

The first of the PaaZ cryoEM maps did not have any bound ligand, which is defined here as the substrate-free structure. The map has an overall nominal resolution of 2.9 Å (Table 1 and Fig. 2a, Supplementary Fig. 2a, b) allowing the tracing of the complete polypeptide chain and assignment of residues after three-fold averaging (Fig. 2b, Supplementary Fig. 2b, c). The model consists of residues 2-679 with the loop regions less well defined. The side chain density for some of the negatively charged residues are poor and the acidic residues were built using the best rotamer that does not create steric overlaps with the nearby residues. Several densities that could correspond to water or ions are observed but have not been modeled. PaaZ is a fusion of N-terminal aldehyde dehydrogenase domain and C-terminal hydratase domain in a single polypeptide. The PaaZ structure containing six monomers can be described as a tri-lobed architecture (Fig. 2a, b). The hydratase domain from three-dimers forms an inner core that holds the whole structure together (Fig. 2c), where each arm or module consists of a domain swapped dimer (Fig. 2d) of PaaZ monomer and the dehydrogenase domain within the dimer is closer to the adjacent hydratase monomer (Figs. 2d and 3a). The peroxisomal multi-functional enzyme (type 2) from the fruit fly (*Drosophila)* is also a domain swapped dimer of enoyl-CoA hydratase and a short chain dehydrogenase-reductase protein^28^. Three short helices (residues 449–459) from each monomer of the hydratase domain form two layers at this inner core (see inset in Fig. 2c). Another helix from each monomer lies perpendicular and wraps around these short helices, forming a tightknit unit. Such an oligomeric assembly is conserved in the structures with enoyl-CoA hydratase fold^29^.

**Table 1:** Cryo-EM data collection, refinement and validation statistics

	PaaZ (EMDB-9873) (PDB 6JQL)	PaaZ + NADPH (EMDB-9874) (PDB 6JQM)	PaaZ + NADP^+^ + OcoA (EMDB-9875) (PDB 6JQN)	PaaZ + NADP^+^ + CCoA (EMDB-9876) (PDB 6JQO)
Data collection and processing				
Magnification	75,000	75,000	75,000	75,000
Voltage (kV)	300	300	300	300
Electron exposure (e^−^/Å^2^)	27	27	27	27
Defocus range (μm)	2.2–3.2	2.2–3.2	2.2–3.2	2.2–3.2
Pixel size (Å)	1.06	1.06	1.06	1.04
Symmetry imposed	C3	C3	C3	C3
Initial particle images (no.)	118,203	175,448	179,312	122,876
Final particle images (no.)	86,420	103,298	101,503	120,968
Map resolution (Å)	2.9	3.3	3.1	3.1
FSC threshold (0.143)^a^				
Map resolution range (Å)^b^	2.5–8	2.5–8	2.5–8	2.5–8
Refinement				
Initial model used (PDB code)	—	—	—	—
Model resolution (Å)	3.5	3.7	3.5	3.5
FSC threshold (0.5)^a^				
Model resolution range (Å)	436–2.9	436–3.3	436–3.1	428–3.1
Map sharpening *B* factor (Å^2^)	98	140	120	102
Model composition				
Non-hydrogen atoms	30,648	30,936	31,278	31,254
Protein residues	4068	4068	4068	4068
Ligands	—	6	12	12
* B* factors (Å^2^)				
Protein	44.4	69	60	44.2
NADP^+^/NAPDH	—	76.5	63.7	45.8
OCoA/CCoA	—	—	53.2	68.9
R.m.s. deviations				
Bond lengths (Å)	0.005	0.006	0.007	0.006
Bond angles (°)	1.2	1.2	1.2	1.2
Validation				
MolProbity score	1.7	1.7	1.7	1.7
Clashscore	6.8	5.8	5.7	5.7
Poor rotamers (%)	0.16	0.06	0	0.13
Ramachandran plot				
Favored (%)	95.2	94.2	94.0	94.6
Allowed (%)	4.8	5.8	6	5.4
Disallowed (%)	0	0	0	0

^a^The Fourier Shell correlation (FSC) value is between two half-maps of the data (@0.143) and the refined model and combined map (@0.5). ^b^from resmap

**Fig. 4:**
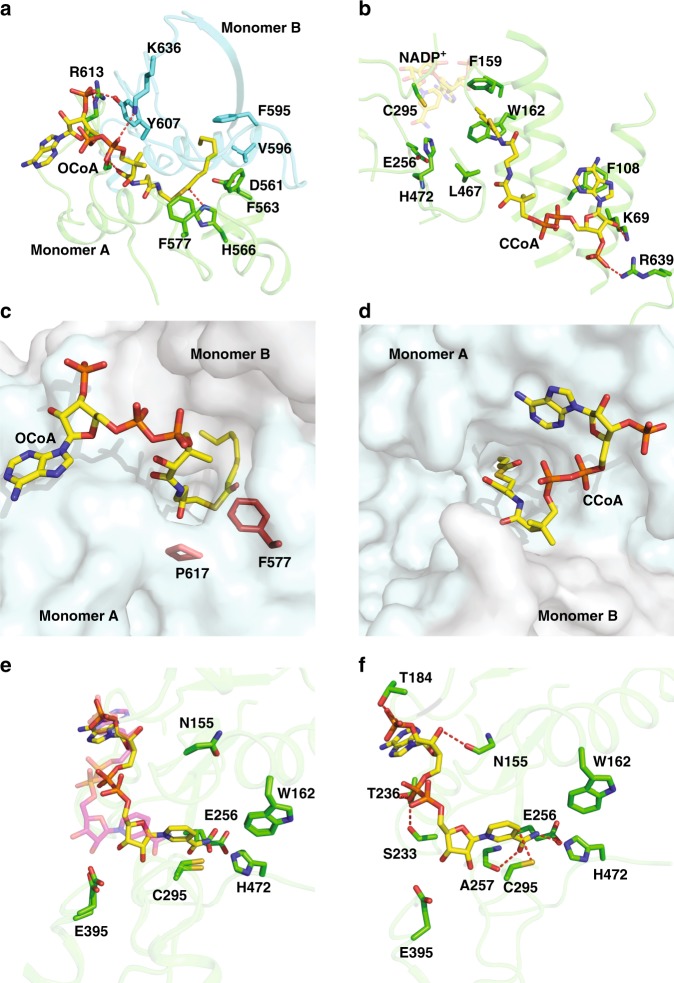
Substrate binding sites in PaaZ. The binding mode and interacting residues are shown for OCoA (**a**) and CCoA (**b**), respectively. The ligands and side chains (carbon atoms in yellow and green color, respectively) are shown in stick representation. **a** OCoA binds at the interface of the two hydratase domains. Residues interacting with OCoA from the individual monomers are colored in green and cyan respectively. **b** The CoA moiety in CCoA interacts with R639, K69 and F108. NADP^+^ and the active site residues are also shown to illustrate how the enzymatic reaction might occur. The nucleophile cysteine (C295) is buried in the core and two aromatic residues F159 and W162 are close to the hydrophobic head group of CCoA. **c**, **d** Surface representation of the enzyme showing the tunnels where the ligands OCoA and CCoA bind. One monomer is colored in cyan and the adjacent monomer in gray for clarity. In the hydratase domain, the head group of OCoA traverses and reaches the adjacent monomer. In the dehydrogenase domain, much of the interactions with the substrate is mediated by one monomer. **e** Overlay of the NADPH/NADP^+^ and the active site of PaaZ + NADPH and OCoA + NADP^+^. The active site residues from both models are shown in stick representation with carbon atoms colored in green. The NADP^+^ and NADPH molecules are shown in yellow and magenta, respectively. The displacement of the E256 side chain as a result of nicotinamide ring is clearly visible while other residues (such as N155, W162, H472) show no change. **f** The interaction of NADP^+^ with the enzyme in the PaaZ-OCoA + NADP^+^ model. The ligand and key residues are shown in stick representation; the carbon atoms of the ligand and the enzyme are shown in yellow and green respectively. Much of the interactions involving the phosphates of the ligand are the same as in the structure of NADPH alone. Due to the position of the ribose and nicotinamide ring close to the active site, the C4 atom is now placed near C295. The ligand is thus stabilized by the hydrogen bonds between the carbonyl and nitrogen atoms of the nicotinamide ring to E256 and H472

The monomer unit consisting of the hydratase and dehydrogenase domain of PaaZ is shown in Fig. 3a. Both these domains of PaaZ share similar folds to the crystal structures of the other dehydrogenase (PDB ID: 2VRO from *Burkholderia xenovorans*) and hydratase domains (PDB ID: 5CPG from *Pseudomonas spp*.), with an overall root mean square deviation of 0.7 and 1.7 Å respectively (Supplementary Fig. 3). The hydratase domain comprises a mixture of α-helices and β-strands that has previously been described as the ‘hot-dog’ fold^30^. Domain swapping results in the active sites of one of the monomer being closer to the substrate-binding site of the adjacent monomer (Figs. 3b and 4c). In the PaaZ structure, the α-helices of the hydratase domain are involved in interactions that form higher-order oligomers and a bundle of β-strands protrude outside, facing the dehydrogenase domain (Fig. 2d). The active site of the hydratase domain inferred from the homologous structures comprises of D561 and H566^24^. The catalytic aspartate is within hydrogen bonding distance to H583 (Fig. 3b). The dehydrogenase domain can be divided into sub-domains consisting of co-factor binding, catalytic and dimerization motifs (Fig. 3c). Each of these sub-domains comprise a mixture of α-helices and β-strands. The active site residues of dehydrogenase domain, also inferred from the homologous structures, comprise the nucleophile C295, and general base E256^23^. Both C295 and E256 can form potential hydrogen bonds to H472 (Fig. 3b). An asparagine residue (N158) is ~5 Å away from C295. However, it is known that the side chain of C295 has some flexibility and can potentially also form a hydrogen bond to N158 in an alternate conformation^31^. The dimerization motif consists of an α-helix and 2 β-strands, which interact with the catalytic domain of the opposite monomer (Fig. 3c).

### Cofactor and substrate binding in PaaZ

One of the main objectives of this study was to understand the molecular basis of substrate transfer from one domain to another in PaaZ. No substrates or cofactors were used during the purification of PaaZ. Structures were determined with substrate analogs and cofactor by cryoEM after simple mixing and incubation with the enzyme followed by grid preparation. The substrate of PaaZ is oxepin CoA, which is not commercially available; instead, ring opened mimics such as octanoyl and crotonyl CoA were used. Structures of PaaZ with (1) NADPH alone, (2) octanoyl Coenzyme A (OCoA) along with NADP^+^ and (3) crotonyl Coenzyme A (CCoA) along with NADP^+^ (Table 1 and Supplementary Figs. 4 and 5) were determined. No major structural changes were observed between these structures. The difference maps illustrating the densities of the bound ligands are shown in Supplementary Fig. 6. In fact, 3D classification of cryoEM data of both substrate-free and ligand bound PaaZ reveals that more than 85% of the particles classify into one single class ruling out any large domain movement. The root mean square deviation (rmsd) of the models derived from the maps of the NADPH or OCoA bound PaaZ to the substrate-free enzyme is 0.4 Å for all the Cα atoms and that of CCoA bound model is 0.95 Å. Of the three ligands described here, the density for cofactor (NADPH/NADP^+^) in all the structures is substantially better allowing for the observation of several interactions with the dehydrogenase domain (Supplementary Fig. 7a–c). Additional densities close to the oxygen of the phosphate were observed. They are likely to be either metal ions or water, but they are not modeled currently. The nicotinamide ring is placed ~4 Å from the active site C295 in the structure of PaaZ with NAPDH alone (Fig. 3d). The carbonyl group of the nicotinamide hydrogen bonds to backbone carbonyl of A257. The co-factor resides on a groove on the top of the dehydrogenase domain with the nicotinamide ring pointing into a cavity that harbors the active site (Fig. 3e). The position and mode of interaction of the NADPH in PaaZ is similar to that in other dehydrogenases^23^.

Substrate mimics OCoA and CCoA used in this study were expected to bind to both the dehydrogenase domain (as the long aliphatic chain is the preferred substrate) as well the hydratase domain (as the ring opened linear form is the product). The first structure obtained with OCoA shows a strong difference density in the hydratase domain (Supplementary Fig. 6b, d). Within each dimer of the PaaZ module, the density for OCoA is continuous in one monomer while in the other monomer the density is continuous till H566 and a short stretch of density is also found downstream of H566 (interaction depicted in Fig. 4a and densities shown in Supplementary Fig. 7d, e). Modeling of OCoA revealed that the CoA moiety from the adenine ring till the sulfur atom could be placed in the continuous density and the terminal 6 carbon atoms in the short stretch leaving two atoms out of the density. It is not clear why the density is fragmented in a region closer to the active site in three of the monomers (Supplementary Fig. 7d, e). We have built the complete model of OCoA in all the monomers. The proposed catalytic residues of PaaZ-hydratase domain D561 and H566 are present on a loop that connects two helices (548–559 and 583–596). D561 and H566 are present at the interface of the two hydratase domains and the side chains point towards the path taken by the hydrophobic head group of OCoA (Fig. 4a). The O1′ atom points towards H566 and is within hydrogen bonding distance. A tunnel can be traced to the core of the hydratase domain with F577 and P617 forming its entrance (Fig. 4c).

The adenine and the phosphate moiety of OCoA are closer to the surface of the protein and exposed to the solvent. The conserved R613 and residues Y607 and K636 from the neighboring monomer interact with the negatively charged CoA, thus, stabilizing the binding of the tail group (Fig. 4a). The hydrophobic moiety is buried in the hydratase domain of PaaZ shielded by residues from adjacent monomers; the residues from both monomers contribute to the structure of the tunnel where the substrate is housed (Fig. 4c), a common feature in hydratases^24^.

In the map of PaaZ-OCoA, another density is observed close to F108 in the dehydrogenase domain. However, it is poorly resolved and could not be modeled. When a map of PaaZ with CCoA was subsequently obtained, the density near F108 was better resolved than the PaaZ-OCoA map and CCoA could be modeled. The density indicates that CCoA unlike NADP^+^ or OCoA is observed at lower occupancy (Supplementary Figs. 6c and 7f) and has some degree of flexibility at the CoA moiety but the density of the hydrophobic head group is better resolved. Similar to the location of catalytic sites on the hydratase site, the nucleophile at the dehydrogenase site C295 is present at the core of the protein, which is buried and accessible through a tunnel (Fig. 4b, d). The hydrophobic head group of the CCoA transverses into the dehydrogenase domain stopping just before the aromatic side chains of W162 and F159, which perhaps provides a path for, or guides the substrate towards the active site C295 (Fig. 4d). Similar to the R613 in the hydratase domain, K69, F108 and R639 form stabilizing interactions with the CoA group of CCoA, which is the substrate analog at the dehydrogenase domain (Fig. 4c). It is not clear if CCoA is also bound to the hydratase domain as the density is ambiguous and no clear difference density is observed.

In both structures of PaaZ with OCoA and CCoA, the position of the NADP^+^ is different from that of PaaZ with NADPH alone. Close to the C4 atom of the nicotinamide ring the density is continuous to active site residue C295 (Supplementary Fig. 7b, c). In the structures with substrate mimics, the nicotinamide ring is placed in the active site by displacing the side chain of E256 such that the distance of the carbonyl oxygen from the sulfur atom of C295 increases to ~6 Å from 3.5 Å (Fig. 4e and Supplementary Fig. 8a). The C4 atom of the nicotinamide ring is 3.5 Å from the C295 and the carbonyl and nitrogen atoms hydrogen bond to the backbone atoms of A257 and side chain of H472 respectively in OCoA and CCoA models (Fig. 4f and Supplementary Fig. 8b). This conformation would allow a mechanism where the pro-R hydrogen position of the C4 atom will point in the direction of the active site. Perhaps, the structure of PaaZ with NADPH alone reflects the state of the enzyme, where the co-factor is leaving after reduction. But due to limited resolution we restrain from making any mechanistic conclusion.

### Substrate Channeling in PaaZ

**Fig. 5:**
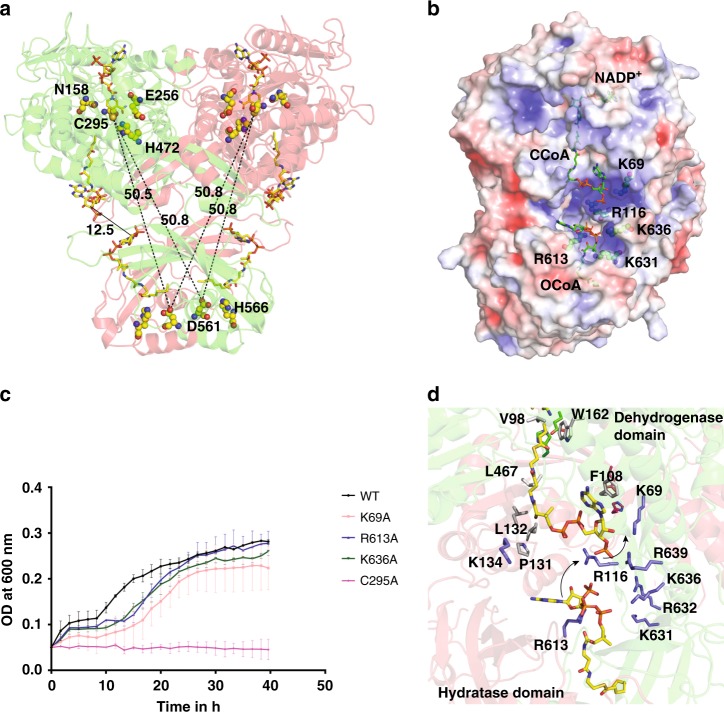
Substrate transfer pathway in PaaZ. **a** Cartoon representation of PaaZ dimer, monomers colored in red and green. The active site residues and ligands are shown in sphere and stick representation, respectively. The distance between the active site residues between the two catalytic centers measured from the same monomer or adjacent monomer is ~51 Å. The ligands OCoA and CCoA from the two structures are used as reference to show the relative positions of the substrates in each enzymatic domain. The distance between the substrates measured from C3 carbon is ~12.5 Å. Note that the OCoA ligand placed in the hydratase domain of the PaaZ CCoA structure is not an experimental structure. **b** Electrostatic (ABPS) potential of PaaZ dimer colored between −5 to 5 kT. The positive patches at this interface region indicate that the substrate from the hydratase domain might be attracted and guided into the tunnel of the dehydrogenase domain. Key positively charged residues are shown in sphere representation. **c** Growth curves of *E. coli* K12 ΔpaaZ transformed with plasmid encoding PaaZ variants—WT, K69A, R613A, K636A and the control C295A. All 3 alanine mutants of the positive charged residues showed slower growth compared to WT, when grown in a minimal media with PA as the sole carbon source. The mutant K69A was the slowest suggesting its role in transfer of substrate from hydratase domain to dehydrogenase domain. Error bars reflect the s.d. of three independent experiments (*n* = 3). The source data are provided as a Source Data File. **d** A close up view of the substrate transfer pathway in PaaZ, colored as in panel **a**. The possible mode of substrate transfer is shown with black arrow. Conserved residue R613 stabilizes the substrate in the hydratase domain and upon ring opening, the substrate could potentially interact with R116. The presence of other charged residues (K636, K631 and R632) are likely to stabilize or guide the substrate toward R116 and the dehydrogenase domain. Subsequently, the hydrophilic tail is stabilized by K69 and only in this step, is the hydrophobic head group likely to be flipped in to the tunnel of the dehydrogenase domain

The distance between the active sites as measured from the side chains of C295 (active site of aldehyde dehydrogenase) and D561 (active site of hydratase), either from the same monomer or different monomers within a dimer, is symmetric and ~51 Å (Fig. 5a). Thus, the possibility that the domain swapping of monomers might bring the active sites of the two enzymatic domains closer does not seem valid. However, the distance between the position of the ligands (OCoA and CCoA) in the swapped monomer is ~12.5 Å (as measured from the C3 carbon of the ribose ring), highlighting the importance of domain swapped architecture. In the absence of a molecular tunnel that connects both the active sites, molecular dynamics (MD) simulation combined with SAXS were employed to investigate if the protein undergoes large domain motions to bring the active sites closer to facilitate the transfer of substrates. The theoretical SAXS profile computed for cryoEM structure fits well with the experimental SAXS profile with a chi value of 4.3. The structure space, sampled for other plausible conformations using BILBOMD, resulted in models with a better fit with experimental SAXS profile (chi value of 2.9). When two of the models that were most prominent (Model A—87% and Model B—13%) were used, the fit was enhanced to a chi value of 2.5 (Supplementary Fig. 9). However, in all of the generated models, the catalytic sites on both domains remain far apart (in the range of 40–50 Å). Thus, even in solution, PaaZ may not undergo large conformational changes required to bring the catalytic sites close enough to facilitate substrate channeling through domain motion.

The tail group of the substrate, Coenzyme A is negatively charged and coincidentally, a complementary positively charged surface can be found at the entrance of both of the domains’ tunnels (Fig. 5b). Pictorially, this is shown using the electrostatic map revealing a surface of positively charged residue at the interface of the domain swapped dimer in each lobe (Fig. 5b). This surface forms a link from the active site of the hydratase domain from one monomer to the entrance of the tunnel of the dehydrogenase domain in the adjacent monomer. This charged surface might ensure that the intermediate does not escape into the bulk solution. We reasoned that the charge in this region between two enzymatic domains act as an attracting point for the CoA moiety from the hydratase domain, followed by the flipping of the hydrophobic head into the channel in the dehydrogenase domain that leads into its active site. A number of charged residues including K69, R116, K636 from one monomer and R613 from the adjacent monomer are in close proximity to the phosphoadenosine moiety of CoA and may mediate the anchoring of the intermediate.

We analyzed the evolutionary conservation of the above residues over 452 homologs of PaaZ using Consurf^32^ and found them to be conserved (Supplementary Fig. 10a). To validate the role of conserved positively charged amino acids in channeling the substrate from hydratase to dehydrogenase domain, we assayed the ability of *E.coli* ΔPaaZ to grow in M63 minimal media with phenyl acetate (PA) as the sole carbon source transformed with plasmids containing PaaZ mutants. Three of these positively charged residues K69, R613 and K636 were mutated to alanine (Ala) and C295, an active site residue of dehydrogenase domain was also mutated to Ala as a control. When compared to wild type PaaZ (wt-PaaZ), all three alanine substitutions of lysine/arginine showed slower growth rates, with the K69A mutant the slowest (Fig. 5c). As expected, the catalytically inactive mutant C295A did not show any growth in minimal media with PA as the sole carbon source. Purified K69A mutant had a similar size exclusion chromatography profile as the wild-type enzyme indicating that the mutation has not affected the protein folding/stability (Supplementary Fig. 10b). These results support our hypothesis on the role of charged residues in channeling of substrate from one enzymatic domain to other.

## Discussion

The goal of this study was to understand how the substrate is transferred (channeled) internally without release into the bulk solvent in the bifunctional enzyme PaaZ, where a hydratase and dehydrogenase enzymatic activity is present in the same polypeptide. Substrate transfer/channeling in enzymes can occur by one of the many ways including the formation of tunnels in the interior of the protein, charged residues guiding the substrate, etc^6^. The occurrence of substrate channeling was first reported in tryptophan synthase where indole, an intermediate, is channeled through a 25 Å long tunnel traversing the protein interior to the next reaction center consequently connecting the two active sites^33^. Other structures such as malate dehydrogenase–citrate synthase complex, and the E1-E2 of pyruvate dehydrogenase complex have also been shown to channel intermediates using intramolecular tunnels^1^. However, such tunnels are not universal. In complexes such as the dihydrofolate reductase and thymidylate synthase, instead of tunnels, positively charged residues along the surface between the active sites were hypothesized to form an “electrostatic highway” sufficient to channel the negatively charged dihydrofolate^34^. Metzger et al., used a combination of Brownian Dynamics (BD) simulation and biochemical experiments to show that the electrostatic highway plays an important role in substrate channeling in DHFR-TS complex from several species^35^.

In the case of PaaZ, it has been previously shown that the product of the hydratase domain is the substrate for the dehydrogenase domain. Additionally, mutation in the dehydrogenase domain alone results in blockage of the pathway^16^. As the substrates are relatively hydrophobic, our first hypothesis was that either a large domain movement that brings the active sites closer or a tunnel is formed between the active sites shielding the substrate from exposure to a hydrophilic environment. The initial estimate of molecular weight by size exclusion chromatography and light scattering indicated that PaaZ forms an oligomer. But the oligomeric state determination using DLS (R_h_ of 8 nm) and SAXS (R_g_ of 6 nm) did not yield unambiguous results as these techniques assume even distribution of atoms within the shape. The exact oligomeric state became evident when PaaZ was observed by cryoEM. The assembly of six monomers of PaaZ into a tri-lobed structure was not intuitive. On hindsight with existing knowledge that dehydrogenase and hydratase often form dimers, the architecture of PaaZ can be appreciated. Although, a domain swapped dimer of PaaZ can in principle form a minimal functional unit, the formation of the trimeric assembly observed here stabilizes the whole complex.

The position of NADP^+^ close to C295 in the dehydrogenase domain and the location of the OCoA in the tunnel of the hydratase domain and close to H566 (Fig. 4a, c, f and Supplementary Fig. 8b) reinforces the identity of the active site residues inferred from the homologous structures^23,24^. Further the location of three ligands in the structures of the enzyme provide a plausible hypothesis on how the substrate might be transferred from one active site to the other. As described in the results section, combination of SAXS studies and MD simulations together rule out any large domain movement in PaaZ. This is also supported by lack of multiple populations when 3D classification was performed with the cryoEM data. The degradation of paa is initiated by ligation of co-enzyme A (CoA) by PaaK resulting in a net negatively charged tail group. The substrate thus has charged polar group and a hydrophobic moiety. In both the hydratase and dehydrogenase domains, the hydrophobic head of the substrate is occluded in the protein whereas the CoA moiety is found interacting with charged and polar residues. The structure also reveals surface of positively charged residues at the interface of the domain swapped dimer, which we predict could act as attracting point for the negatively charged CoA to interact (Fig. 5b).

Indeed, we find that the positive charged residues (K69, R116, R613, K631 and K636) are highly conserved in PaaZ homologs (Supplementary Fig. 10a). Our analysis on few of these positive charged mutants of PaaZ for their ability to provide growth in media containing PA as the sole carbon source supports the idea of this region acting as a conduit for substrate transfer (Fig. 5c). Interestingly, several bacterial species contain gene products for the individual domains of aldehyde dehydrogenase and enoyl-CoA hydratase in addition to containing gene product encoding for a fused PaaZ homolog. When twenty such sequences representing gene products of individual aldehyde dehydrogenase, enoyl-CoA hydratase and fused PaaZ homologs were analyzed, only residue R613 was conserved in all the sequence and the other four positively charged residues are conserved only in the fused PaaZ homologs (Supplementary Fig. 10b). The domain swapped arrangement ensures the distance between the substrate tunnels of the two enzymatic domains are close. The orientation also enables several positively charged residues from the swapped chains to come close (within the dimeric unit) and form a positively charged anchor surface. The presence of multiple charged residues perhaps provides redundancy and mutating all these residues might greatly impair the substrate transfer, while modifying any one will only reduce the efficiency (Fig. 5c).

One potential issue is the possibility of the substrate getting trapped in the cluster of the positive residues during its transition from the hydratase to the dehydrogenase domain. But the presence of the hydrophobic head group in the substrate might drive the interaction with positive charged residues to be transient (Fig. 5d). From the current model, it is evident that the phosphoadenosine moiety of the substrate (OCoA) anchored through its interaction with R613 of the hydratase domain will be attracted towards the R116 of the dehydrogenase domain of the adjacent monomer. K636, which forms part of the cluster that includes K631 and R632 might stabilize this transition and as the distance to R116 from R613 is short, the hydrophobic head group could still be in the tunnel of the hydratase domain (Fig. 5d). In the next step, the substrate can then migrate towards K69 and R639, which can stabilize the CoA moiety. Thus, the hydrophobic head of the substrate doesn’t have to get exposed or travel far and is flipped into the tunnel of the dehydrogenase domain.

This hypothesis is analogous, but not identical, to that of the fatty acid β-oxidation multi-enzyme complex, where a single site binds to the CoA and acts as a pivot for transfer of the intermediate between the two active sites^5^. Intramolecular tunnels might seem to be an obvious choice for the channeling mechanism as they may prove beneficial when the intermediate is uncharged and needs to be protected from exposure to solvent. However, as described in the DHFR-TS complex, electrostatic surfaces would likely be of greater generality, for the reason that most metabolic intermediates are charged^34^. In substrates, where part of the structure is significantly polar and part hydrophobic, a more likely mechanism would involve electrostatic pivots or anchors, followed by movement of the hydrophobic part from one active site to another. Our structural studies on PaaZ reported here suggests such a mechanism. However, contrary to the earlier observed mechanism for pivoting, our SAXS experiments and theoretical simulations do not suggest any significant conformational change. The conserved set of positively charged residues might play a critical role in pivoting and the transfer of intermediates from the one subunit to another in PaaZ.

## Methods

### Protein purification

The gene (*PaaZ*) was amplified from *E. coli* K12 reference genome using the following primers -TACCCATGGGCCATCATCATCATCATCACCAGCAGTTAGCCAGTTTCTTATC (forward) and CGTCTCGAGTTAATCGACAAAATCACCGTG (reverse). The PCR product was cloned into pET28a plasmid using the NcoI and XhoI restriction sites. The protein was expressed in *E.coli* BL21 DE3* (Invitrogen, Thermo Fisher Scientific) cells in SOC media with 20% glucose at 37 °C. The cells were induced with 100μM IPTG and grown at 18 °C (post induction) for 16 h and harvested. The cells were lysed by sonication in sodium phosphate buffer followed by affinity purification with Ni-NTA beads (Thermo Fisher Scientific). The eluted protein from affinity chromatography was dialyzed overnight in buffer containing 100 mM HEPES, pH 7.4 and 200 mM NaCl. The protein was concentrated using a 30-kDa cutoff centrifuge concentrator (Millipore) followed by size-exclusion chromatography (SEC) using a Superdex 200 preparative column (GE Life Sciences) equilibrated with buffer containing 25 mM of HEPES (pH 7.4) and 50 mM NaCl. The protein eluted at 60 ml corresponding to a molecular weight of ~570 kDa (*n* = 7.8) based on calibration with monodisperse standards (BioRad catalog # 1511901).

### Dynamic light scattering

The peak fractions from the Superdex 200 SEC run were pooled and concentrated to 4.5 mg/ml. Sample monodispersity and hydrodynamic radius was determined at 25 °C using a DynaPro Nanostar dynamic light scattering (DLS) instrument (Wyatt Technology). The data were analyzed using the Dynamics 7.1.7 software.

### Small-angle X-ray scattering

SAXS data were collected on the SIBYLS beamline (beamline 12.3.1) at the Advanced Light Source, Lawrence Berkeley National Laboratory. The SAXS data were collected at 10 °C with three concentrations (1.5, 3.0 and 4.5 mg/ml) in the order of lowest to highest concentration with increasing exposures of 0.5, 1, 2 and 5 s each. Scattering from the buffer (matched against the samples by overnight dialysis) was subtracted and the sample scattering data analyzed by Primus^36^. No concentration-dependent effects were observed in the low-q region on comparison of the low exposures for each concentration. However, detector saturation in the low-q region was observed at higher exposures for all concentrations. Based on this analysis, a merged data set was created from the highest concentration using the low-q regions of the low exposure scattering curves and the high-q regions at the highest exposure. AUTORG and DATGNOM^37^ were used to calculate R_g_ (radius of gyration) and Dmax (maximum particle dimension from a pairwise distribution function) in PRIMUS and ab inito envelopes were generated using the GASBORI^38,39^, GASBORP, and DAMMIF^40^ programs in the ATSAS 2.4.2-1 package. Eight ab initio envelopes generated independently using the above programs were averaged/filtered using DAMAVER^41^ and aligned to the cryoEM structure using SUPCOMB^42^.

### Electron microscopy and image processing

The initial data sets of PaaZ were collected using normal Quantifoil holey carbon grids with blotting and freezing accomplished with a manual plunger. These grids were made with a protein concentration of 1–2 mg/ml. Though there was a sufficient number of particles in each image, the protein showed tendency to clump together on the cryoEM grid. These initial data were collected with Titan Krios and Falcon 2 detector in integration mode with the EPU software (ThermoFisher-FEI). The class averages from this data set were used to generate an initial model using EMAN2^43^. It was possible to obtain ~4 Å resolution with 65,000 particles using a sampling of 1.75 Å/pixel and C3 symmetry imposed. Protein clumping on cryoEM grids proved to be an impediment. Clumping restricted the ability to obtain higher-resolution maps and attempts to overcome this problem by changing the pH and the use of detergent were not successful.

Subsequently, the use of graphene oxide on Ultragold grids gave excellent distribution with very little clumping. The preparation of graphene oxide was done as described previously by Bokori-Brown et al.^44^ with the following modifications. UltraFoil 1.2/1.3 300 mesh grids were rendered hydrophilic by glow-discharging for 5 min at 0.2 mBar and 40 mA current using an Edwards sputter coater S105B operating in glow-discharge mode. The longer glow discharge allowed for more graphene oxide to attach. 2% (2 mg/ml) of Sigma GO (cat. # 763705) in water was diluted to 0.2% working solution. Diluted 0.2% working solution was spun down for 1 min at 100 × *g* before grid preparation. 3 μl of 0.2% graphene oxide was placed on the grid and left to stand for 1 min. The fluid was blotted against Whatman filter paper #1 and the grids were then washed with 4 μl of distilled water two times on the side where the graphene was applied and once on the opposite side. Residual fluid was blotted against Whatman paper #1 and grids were left to dry for minimum 4 h before using them. The squares covered with graphene oxide are easily identified using the low mag (atlas/grid square image) with the EPU software. Within the squares, on average 70% of holes had graphene oxide and on Ultrafoil Au grids, distinguishing holes with or without graphene oxide is not easy. Invariably, we did observe some images that had no graphene and hence no particles. In a typical dataset, 65–70% of collected images had good particle distribution and were selected for processing.

For the grids coated with graphene oxide, PaaZ at 0.015 mg/ml was applied and blotted with the Vitrobot for 3 s and plunge frozen into liquid ethane. For datasets with ligand, PaaZ was mixed with respective ligands (10-fold excess to the enzyme concentration) and incubated for 15 min at room temperature followed by plunge freezing as above. All the imaging of PaaZ in graphene oxide was done with EPU and Falcon 3 detector in counting mode at 1.06 or 1.04 Å/pixel. The images were exposed for 60 s with a total accumulated dose of ~27 e^−^/Å^2^ and dose fractionated into 75 frames, with each frame having a dose ~0.3 e^−^. Motioncor2^45^ and or Unblur^46^ were used for the full frame alignment with frames grouped in 3 resulting in 25 frames and ~0.9e^−^/frame (use of Motioncor2 or Unblur gave similar resolutions and maps). Processing was done in Relion 2.0^47^. The summed images were then used for automated particle picking with Gautomatch (http://www.mrc-lmb.cam.ac.uk/kzhang/) with template derived from previous data collection and CTF was estimated with Gctf^48^. Particles were extracted with a box size of 412 pixels and subjected to 2D classification, 3D auto-refinement, per particle motion-correction, B-factor weighting and refinement. This resulted in maps in resolutions between 2.9 and 3.3 Å for the different data sets (Table 1). Further 3D classification was used to improve the quality of the maps by removing bad particles. Difference maps were calculated with final unsharpened combined map (i.e., before post-processing). Local resolution of the maps was estimated with Resmap^49^.

### Model building and refinement

The monomer of the aldehyde dehydrogenase domain from *Burkholderia xenovorans* (PDB:2VRO sharing sequence identity of 43% with PaaZ-Aldh domain)^23^ and the hydratase domain from *Pseudomonas spp*. (PDB:5CPG sharing sequencing identity of 32% with PaaZ-hyd domain)^29^ were manually docked into the EM map using Chimera^50^ after long loop regions deleted. The overall secondary structural fold of both these domains fit well into the PaaZ map. Subsequently, the sequence was converted to alanine and was followed by manual model building and assignment of the residues with side chain density as markers using Coot^51^. Ligands were added to the model after all the residues of the protein were built. The model was refined real space refinement in Phenix^52^. Figures were made with Pymol^53^ and Chimera.

### Molecular dynamics simulation

To understand the protein structure dynamics, BILBOMD^54^ was used which performs rigid body modeling by molecular dynamics simulation and uses a Minimal Ensemble Search (MES) genetic algorithm for identifying the minimal ensembles. BILBOMD uses the CHARMM force field for MD simulation at high temperature to sample the entire confirmation space. The experimental SAXS data is used by BILBOMD to fit the models generated from MD simulation. BILBOMD was performed with an R_g_ range of 54 to 70 nm (where the experimental R_g_ was 60.1 nm). Residues 512–524, which form the inter-domain linker region, were kept flexible and 400 models were generated for each R_g_ (a total of 2400 models).

### Search for sequence homologs

PaaZ protein sequence was used as the query to search against NR database. PSI-BLAST with 3 iterations and an evalue of 0.001 was used for sequence search. The hits obtained were queried against PFAM domain database using hmmscan with gathering threshold. True positive hits which contained both Aldehyde dehydrogenase and Enoyl-CoA hydratase domains in the same sequence were chosen for further analysis. Additionally, BLAST was used to search for gene products that contain only Aldehyde dehydrogenase or Enoyl-CoA hydratase domains in representative genomes with an evalue of 0.001 using PaaZ sequence as query sequence.

### Mutagenesis and growth kinetics

Mutagenesis of PaaZ was performed to determine the role of conserved positively charged amino acids in substrate channeling. PaaZ cloned in PCA24N vector with N-terminal histidine tag, part of the ASKA library was used as the template for all the Site Directed Mutagenesis (SDM)^55^. Whole-vector PCR was performed using Phusion Polymerase (ThermoFisher Scientific) with individual set of primers containing the mutated residue. The primers used for mutagenesis are as follows:

K69A—GGCGATGCTTGCAGCGGTCGCTAAACATC (forward) and GTTTAGCGACCGCTGCAAGCATCGCCGCACGTTC (reverse)

R613A—GGAAAGCTTGGCTTTTATCGAACCCGTAAAG (forward) and GGTTCGATAAAAGCCAAGCTTTCCAGCCCGTAG (reverse)

K636A—GTAAGCGCAAGACGCTGGCAAAACAGCGTAGCGCAG (forward) and CTGCGCTACGCTGTTTTGCCAGCGTCTTGCGCTTAC (reverse)

C295A—CAAAAGCCGGGCAAAAAGCTACGGCAATCCGGCGGATTATTG (forward) and CAATAATCCGCCGGATTGCCGTAGCTTTTTGCCCGGCTTTTG (reverse)

The mutation was confirmed by sanger sequencing. To perform growth kinetics, chemically competent cells of *E.coli* K-12 ΔPaaZ strain, part of the ASKA library was prepared (Note that, *E.coli* K12 uses the hybrid pathway for paa catabolism). The growth kinetics was performed by transforming individual plasmids containing PCA24N-PaaZ Wild Type (WT), PCA24N-PaaZ K69A, PCA24N-PaaZ K631A, PCA24N-PaaZ K636A, PCA24N empty vector or PCA24N PaaZ C295A in *E.coli* K-12 ΔPaaZ strain. The cells were grown in minimal medium M63 supplemented with either Phenylacetate (PA) (10 mM) or Glycerol (5 mM) as sole carbon source with 100 μM of IPTG for inducing protein production^56^. For cells containing plasmids PCA24N-PaaZ Wild Type (WT), PCA24N-PaaZ K69A, PCA24N-PaaZ K631A, PCA24N-PaaZ K636A, M63 media supplemented with 10 mM PA as sole carbon source was used. Since cells containing plasmids PCA24N empty vector or PCA24N PaaZ C295A are auxotrophs to PA, the primary cultures for these two clones were grown in M63 media supplemented with 5 mM Glycerol as carbon source. All the primary cultures were grown for a period of 48 h. M63 media with PA as sole carbon source was used for secondary cultures of all the clones, where the cells were diluted to 0.05 Optical density (OD) to a final volume of 2 ml and growth kinetics was performed in a 12 well plate using Tecan Infinite M200 plate reader. OD at 600 nm was taken every 30 min with orbital shaking. A total of 3 independent readings were collected to plot the growth kinetics. For protein purification, WT-PaaZ (PCA24N vector) and K69A-PaaZ were grown in Luria-Bertani (LB) media at 37 °C and induced with 100 μM IPTG at 0.6 OD. Subsequently, cells were further grown at 18 °C for 16 h and harvested. The mutant and the wild-type enzymes were purified using Ni-NTA chromatography followed by Gel Filtration Chromatography (GFC) as described above. Both enzymes eluted at 60.18 volume on a GE Superdex 200 16/600 column (Supplementary Fig. 9a) suggesting no change in state of oligomerization in K69A-PaaZ mutant.

### Reporting summary

Further information on research design is available in the Nature Research Reporting Summary linked to this article.

## Supplementary information

Supplementary Information

Reporting Summary

Source Data

## Data Availability

The cryoEM Maps and coordinates of PaaZ have been deposited with the EM data bank and the Protein Data Bank with the following codes: Substrate-free (EMDB-9873, 6JQL), NADPH (EMDB-9874, 6JQM), OCoA (EMDB-9875, 6JQN) and CCoA (EMDB-9876, 6JQO). The SAXS data of PaaZ has been deposited in SASDBD under accession code SASDGL2. The source data underlying Fig. 5c is provided as a Source Data File. All other data are available from the corresponding authors on reasonable request.
