# Supplementary material for: Molecular basis for metabolite channeling in a ring opening enzyme of the phenylacetate degradation pathway
Source: Nat Commun. 2019 Sep 11;10:4127. doi: 10.1038/s41467-019-11931-1 (PMC6739347; doi:10.1038/s41467-019-11931-1)
Supplement: Supplementary file 1 — Supplementary Information [file 41467_2019_11931_MOESM1_ESM.pdf]

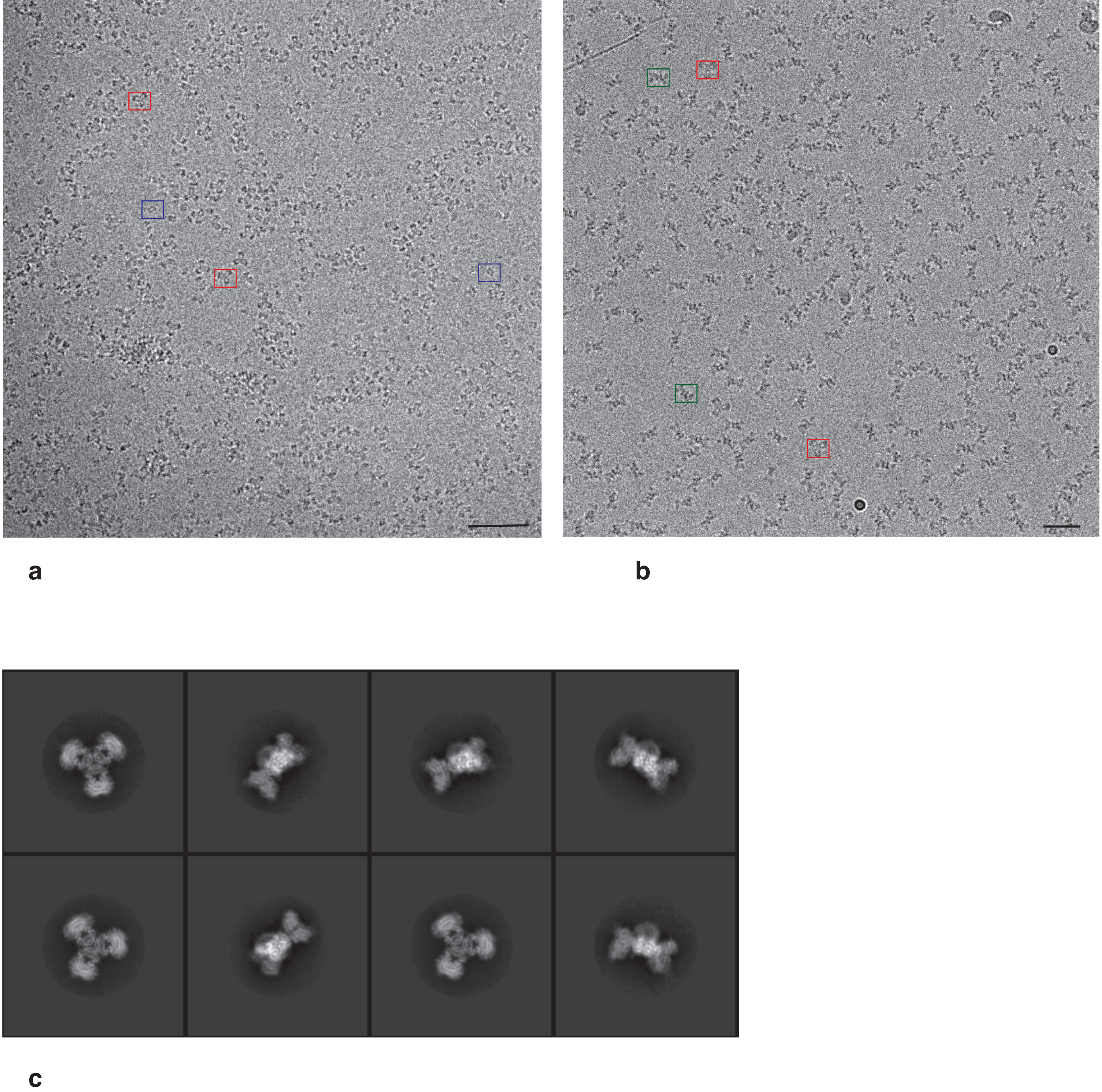

Supplementary Figure 1: CryoEM micrographs and class averages of PaaZ

a Micrograph of PaaZ on ice. Trimeric shape of enzyme (top and bottom view) is marked in red. The occasional dissociated dimer molecules are shown in blue. Scale bar is 800 Å.

b Micrograph of PaaZ on Ultrafoil gold grids coated with graphene oxide. Trimeric shape of PaaZ (top and bottom view) is marked in red. Elongated structures (side view) are marked in green. Scale bar is 300 Å.

c 2D class averages of PaaZ showing the views of the molecule. The box size is 416 pixels and sampled at 1.07 Å/pixel. The class averages shown here were obtained with data collected at the National CryoEM facility, Bangalore.

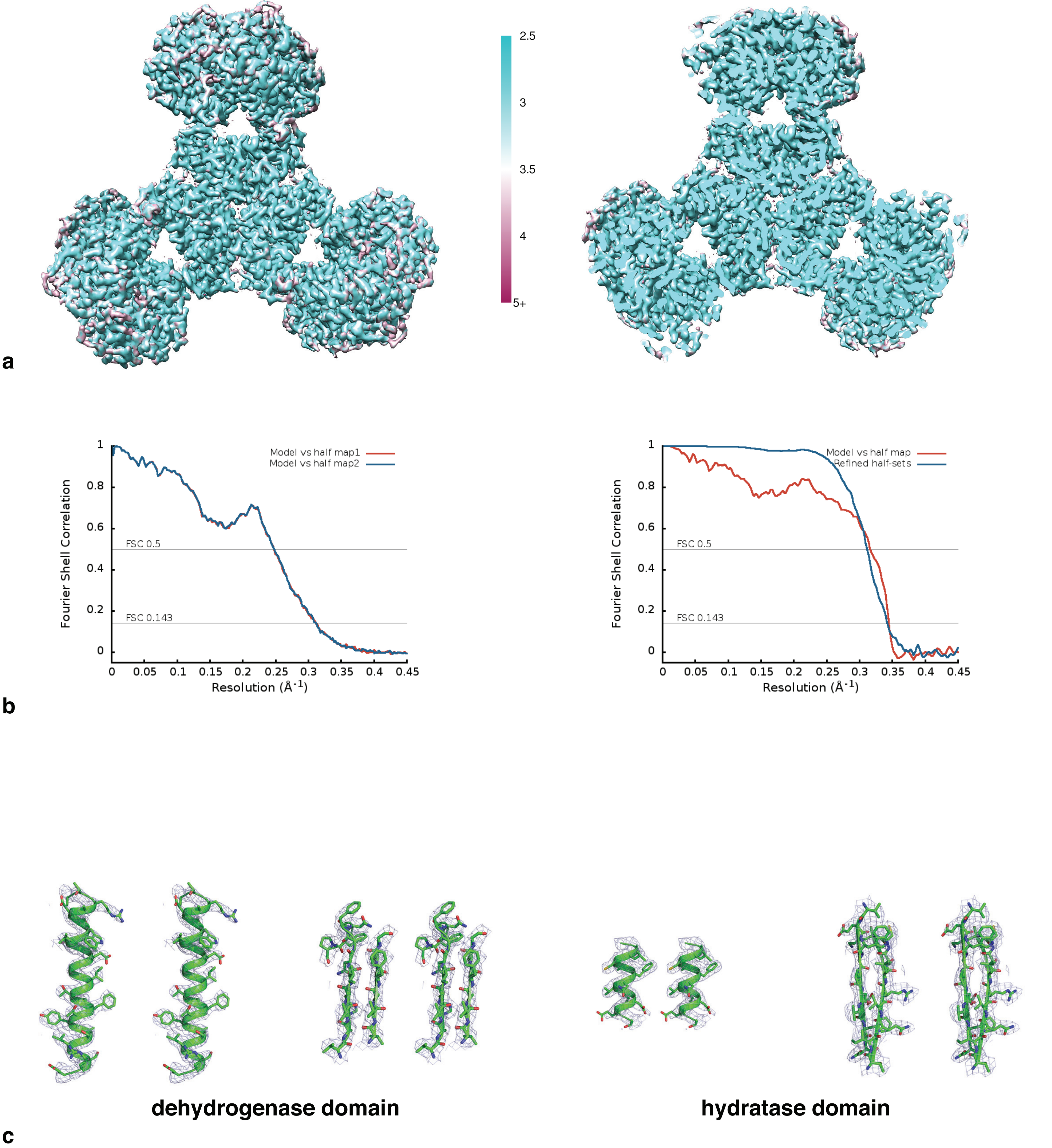

Supplementary Figure 2: **Local resolution plots, FSC curves and density maps of substrate-free PaaZ**

a the local resolution plots of substrate-free PaaZ EM map showing the whole molecule on right and a cut away section on left.
b the refinement of the PaaZ model against one of the half-maps is used to verify overfitting. On the left, the Fourier shell correlation (FSC) curves of two half-maps (blue) and the map and model (red) are shown. The resolution estimated from the half-maps at FSC 0.143 is 2.9 Å and that estimated from map and model at FSC 0.5 is 3.5 Å.
c Examples of EM potential density (grey mesh) is shown for α-helices and β-sheets in stereo view. In the dehydrogenase domain, residues 92-116 (α-helix) and 150-156,227-231 (β-sheet) are shown. In the hydratase domain, residues 548-560 (α-helix) and 623-631,647-656 (β-sheet) are shown. The maps were contoured at 7.0σ and carved 2.0 Å around the atoms. The final B-factor sharpened map was used for making this figure with Pymol.

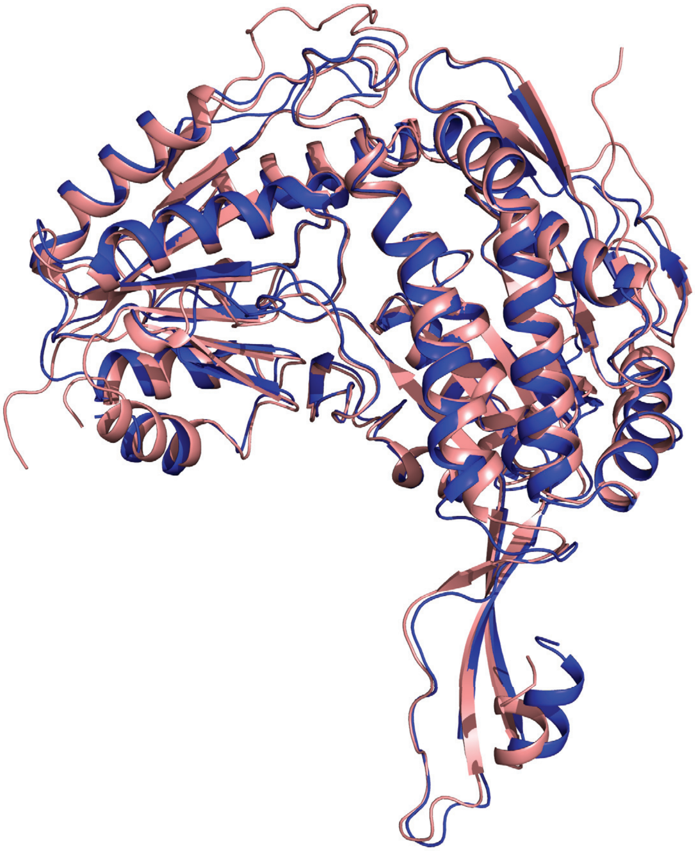

Dehydrogenase domain

PaaZ - blue
2VR0 - salmon

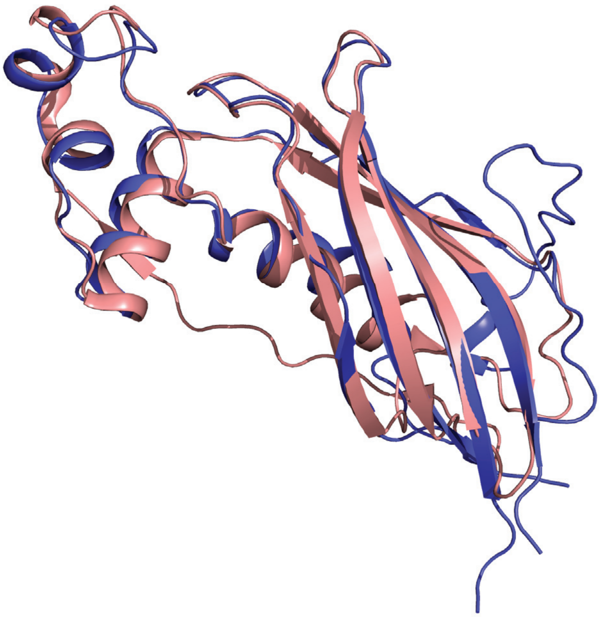

Hydratase domain

PaaZ - blue
5CPG - salmon

Supplementary Figure 3: **Overlay of individual dehydrogenase and hydratase domains of known structures with PaaZ**

The aldehyde dehydrogenase domain from *Burkholderia xenovorans* (2VRO) is shown in salmon and the PaaZ dehydrogenase domain in blue. The RMSD between the molecules is 0.7 Å for the Cα atoms. The enoyl-Coenzyme A hydratase from *Pseudomonas spp.* (5CPG) is shown in salmon and the PaaZ hydratase domain in blue and has an overall RMSD of 1.7 Å for the Cα atoms. The structures of the dehydrogenase and the hydratase domains used for comparison have been determined by X-ray crystallography.

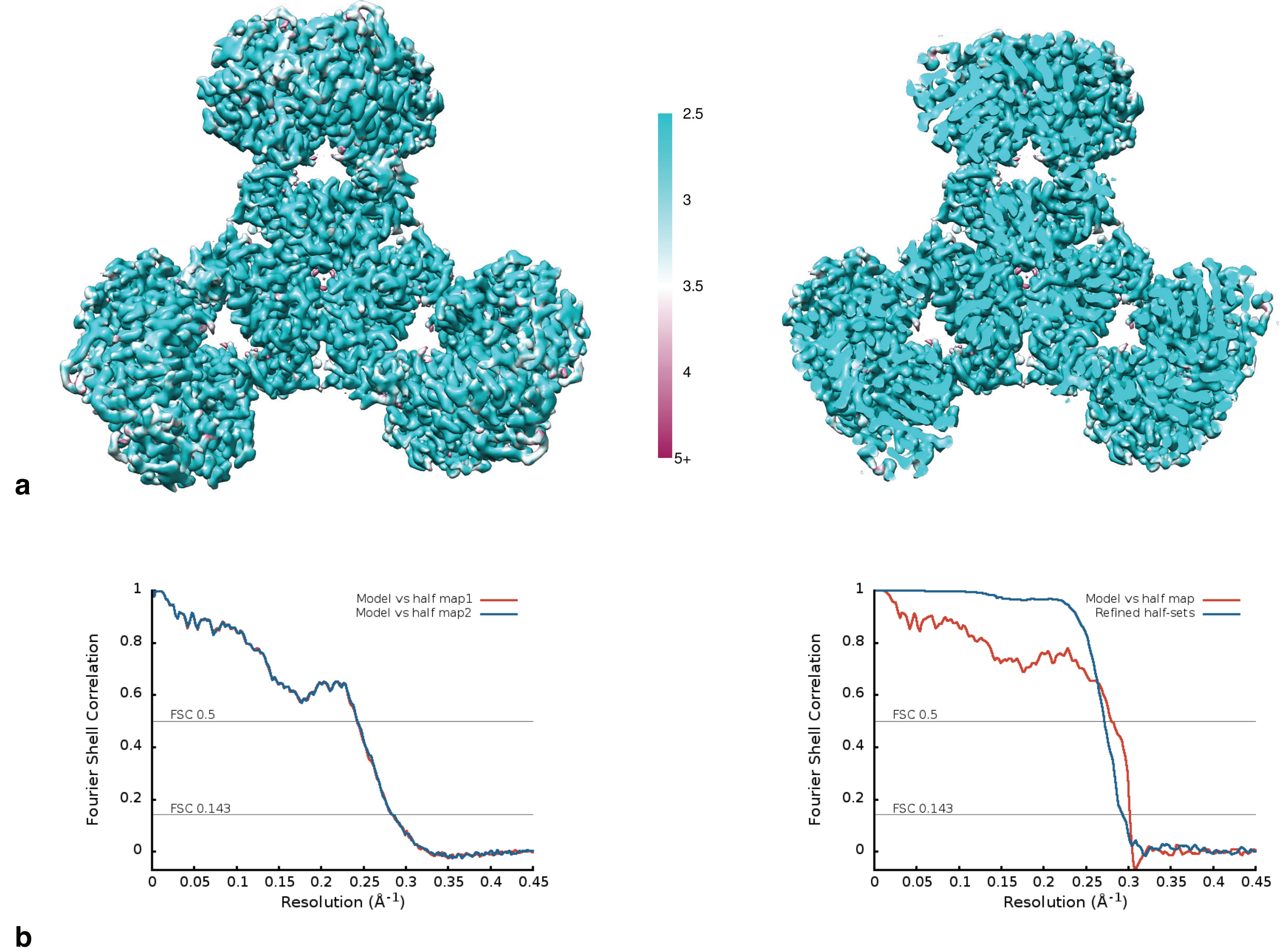

Supplementary Figure 4: **Local resolution plots and FSC curves of PaaZ with NADPH**

a the local resolution plots of PaaZ with NADPH showing the whole molecule on right and a cut away section on left.
b the refinement of the PaaZ+NADPH model against one of the half-maps is used to verify overfitting. On the left, the Fourier shell correlation (FSC) curves of two half-maps (blue) and the map and model (red) are shown. The resolution estimated from the half-maps at FSC 0.143 is 3.3 Å and that estimated from map and model at FSC 0.5 is 3.7 Å.

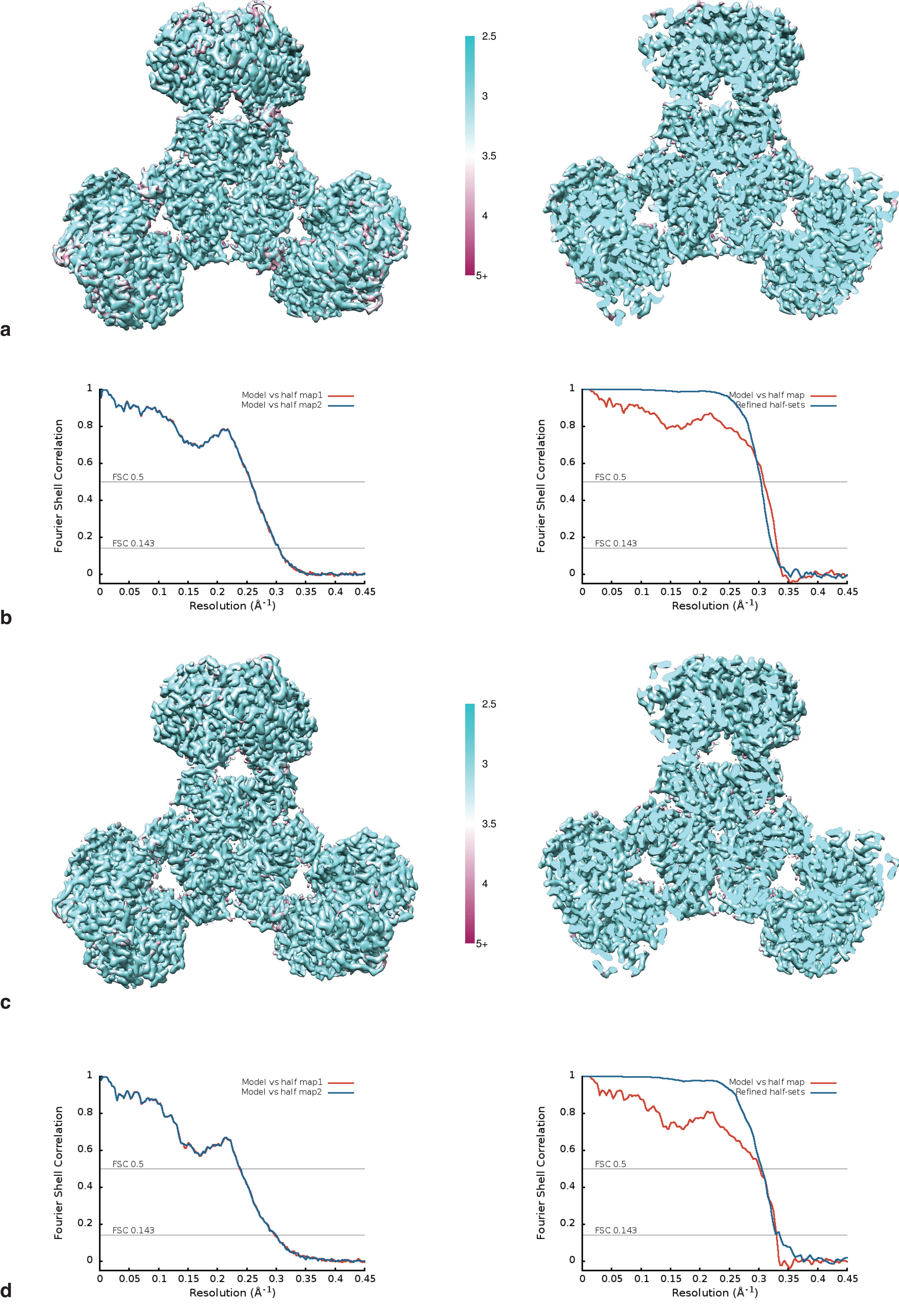

Supplementary Figure 5

Supplementary Figure 5: **FSC curves and Local resolution plots of PaaZ OCoA and CCoA**

a the local resolution plots of PaaZ with $NADP^+$ and octanoyl CoA showing the whole molecule on right and a cut away section on left.
b the refinement of PaaZ OCoA model against one of the half-maps is used to verify overfitting. On the left, the Fourier shell correlation (FSC) curves of two half-maps (blue) and the map and model (red) are shown. The resolution estimated from the half-maps at FSC 0.143 is 3.1 Å and that estimated from map and model at FSC 0.5 is 3.5 Å.

c the local resolution plots of PaaZ with $NADP^+$ and crotonyl CoA showing the whole molecule on right and a cut away section on left.
d the refinement of PaaZ CCoA model against one of the half-maps is used to verify overfitting. On the left, the Fourier shell correlation (FSC) curves of two half-maps (blue) and the map and model (red) are shown. The resolution estimated from the half-maps at FSC 0.143 is 3.1 Å and that estimated from map and model at FSC 0.5 is 3.5 Å.

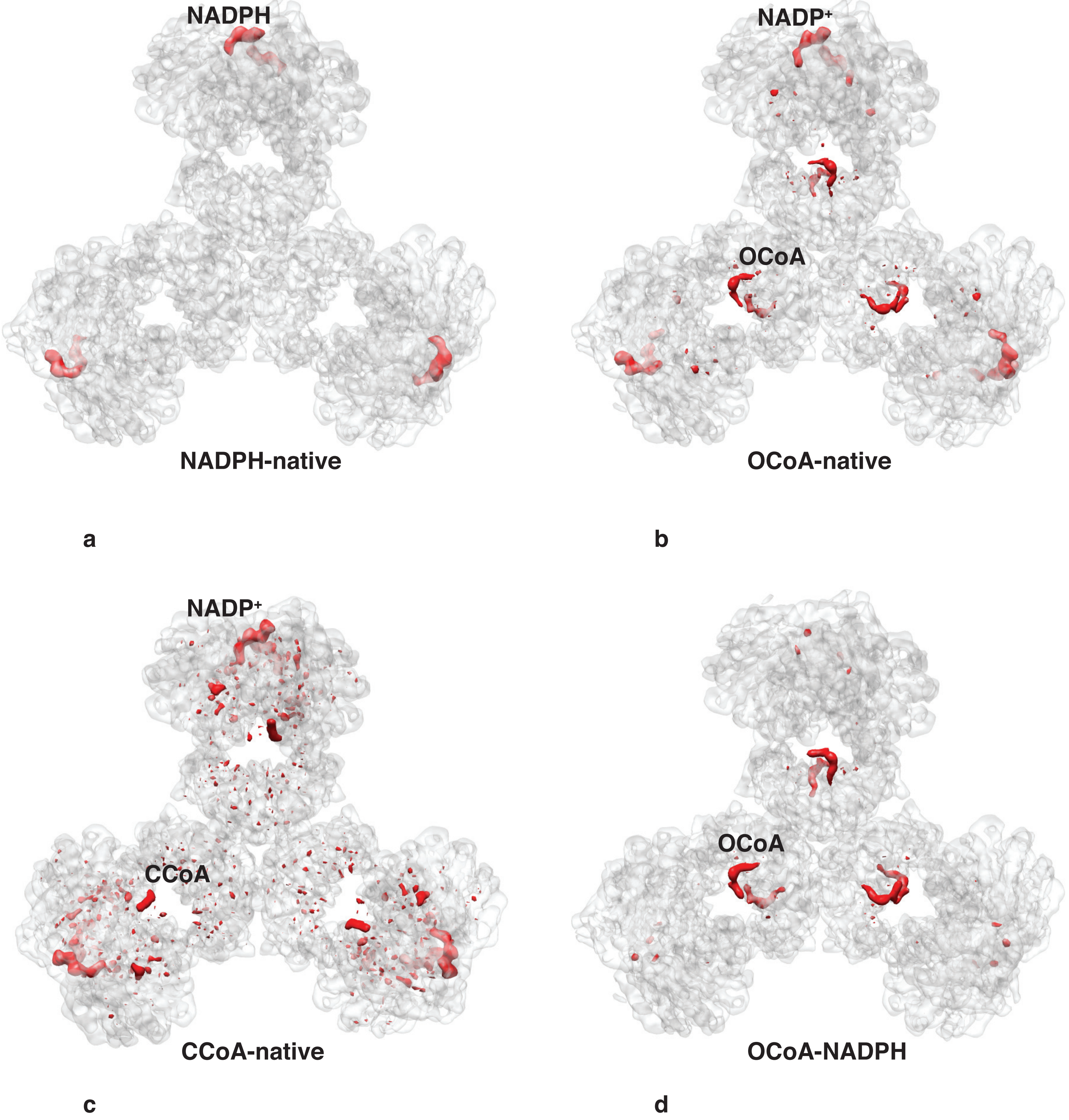

Supplementary Figure 6: Difference maps calculated between the structures of PaaZ

Unsharpened combined maps after refinement were used for the calculation of the difference maps in Chimera. The maps were aligned before the calculation of the difference maps. The PaaZ molecule is shown in transparent gray and the difference density in red. For simplicity, the substrate-free enzyme is called 'native' enzyme.

In panel **a**, the difference between PaaZ NADPH-native is shown and the difference density located in the dehydrogenase domain. In panel **b,** the difference between the $NADP^+$ and octanoyl coA (OCoA) bound PaaZ and the native structure is shown. Note that the two peaks are in distinct domains, the $NADP^+$ in the dehydrogenase domain and the octanoyl CoA in the hydratase domain. The shape of the octanoyl CoA adopted in binding of PaaZ is clearly visible.

In panel **c**, the difference between the $NADP^+$ and crotonoyl coA (CCoA) bound PaaZ and the native structure is shown. As the maps of the native enzyme and the CCoA were obtained from different microscopes, the maps were aligned and pixel size adjusted to get the higest correlation and then difference were calculated. Perhaps due to lower occupancy of the CCoA, the difference density is not as clear as those observed for $NADP^+$ or OCoA but strong peak close to the tunnel (marked with CCoA) is visualised. In Panel **d**, the difference between the PaaZ with $NADP^+$-octanoyl CoA and PaaZ with NADPH only is shown. Here only the peak from OCoA is observed.

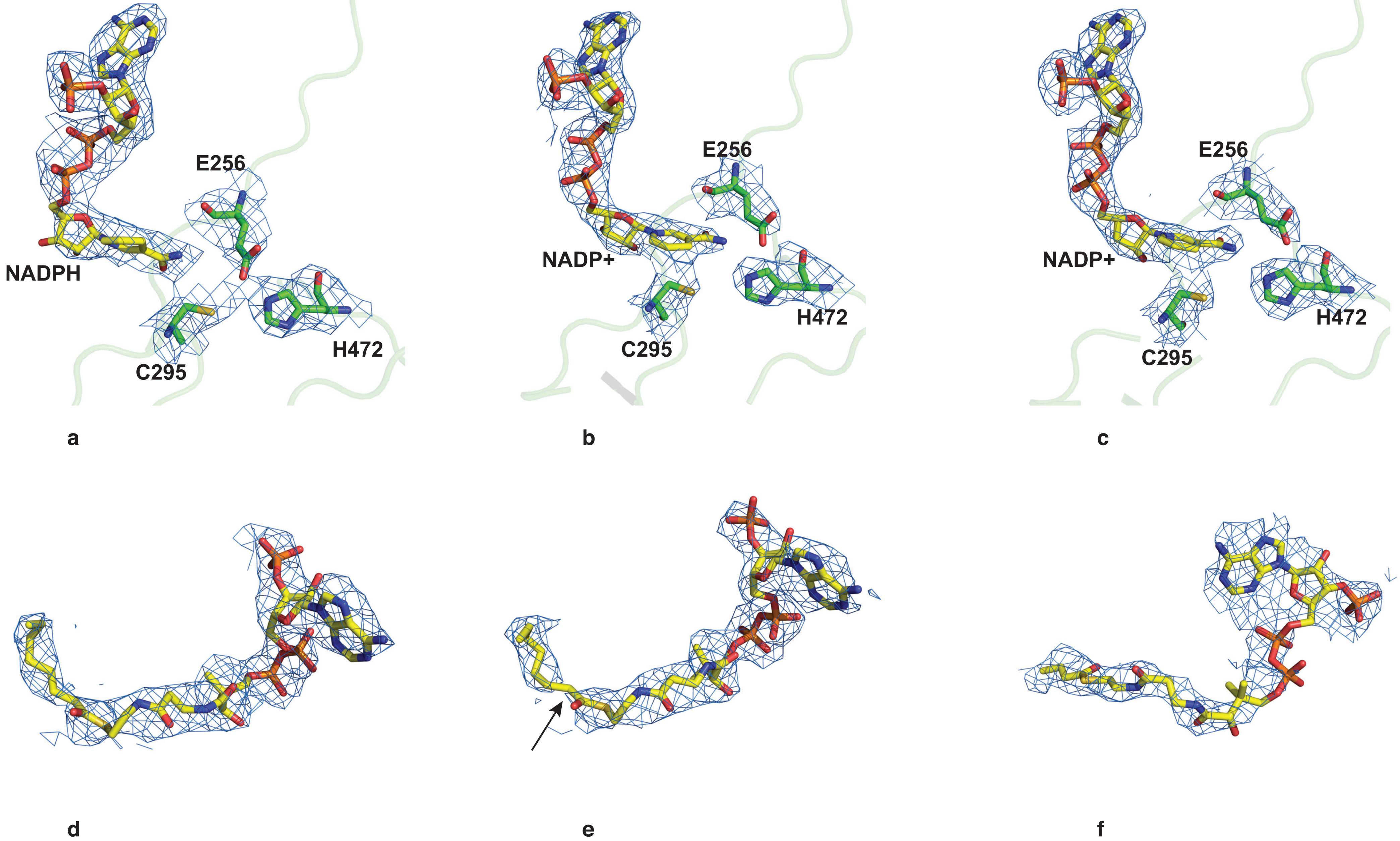

Supplementary Figure 7: **Density of ligands and cofactor in PaaZ reconstructions**

a, b & c Density of NADPH/$NADP^+$ in the maps of PaaZ mixed with NADPH alone or with OCoA and CCoA respectively. The density for active site residues (C295, E256, H472) are also shown. The density in OCoA and CCoA maps show a continuous density from C295 to the $NADP^+$ ligand. In the maps with NADPH alone there is some residual density around the active site residues. **d** Density of OCoA in a monomer where no break in density is observed. **e** OCoA density in a monomer where the break in density is observed (marked with arrow). It is not clear why the density is fragmented in a region closer to the active site in three of the monomers in the PaaZ-OCoA maps. One possibility could be the small differences in the B-factor sharpening between the monomers in the molecule. The density is continuous till the active site H566 and a short stretch of density is also found downstream of H566. **f** Density of CCoA. The final sharpened maps were used to make the figures with Pymol and the maps have been carved around 2 Å of atoms.

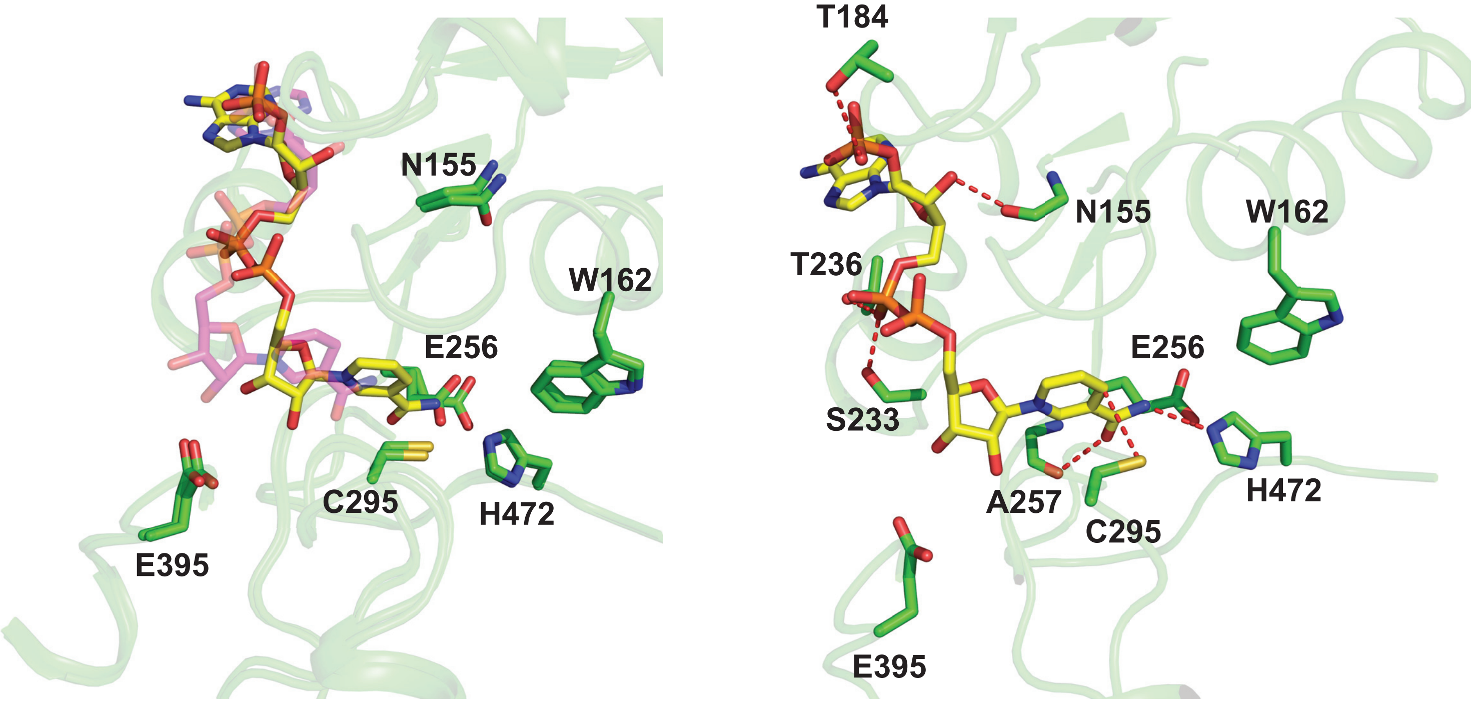

a **b**

Supplementary Figure 8: The active site of PaaZ with CCoA and NADP⁺

a An overlay of the NADP⁺/NADPH and the active site of PaaZ+NADPH with the model of CCoA+NADP⁺. The active site residues from both the models are shown in stick representation (carbon atoms in green). The NADP⁺ and NADPH molecules in the models of PaaZ CCoA+NADP⁺ and PaaZ+NADPH are coloured in yellow and magenta respectively. The displacement of the E256 side chain as a result of nicotinamide ring is clearly visible. There are no significant changes in other residues in the active site (C295 and H472) or other residues not involved in catalysis such as N155 and W162.

b The interaction of NADP⁺ with the enzyme in the PaaZ-CCoA+NADP⁺ model. The ligand and key residues are shown in stick representation; the carbon atoms of the ligand and enzyme are shown in yellow and green respectively. Much of the interactions involving the phosphates of the ligand is the same as in the structure of NADPH alone. Due to the position of the ribose and nicotinamide ring, the C4 atom is now placed closed to C295. The ligand is now stabilized by the hydrogen bonds between the carbonyl and nitrogen atoms of the nicotinamide ring to E256 and H472.

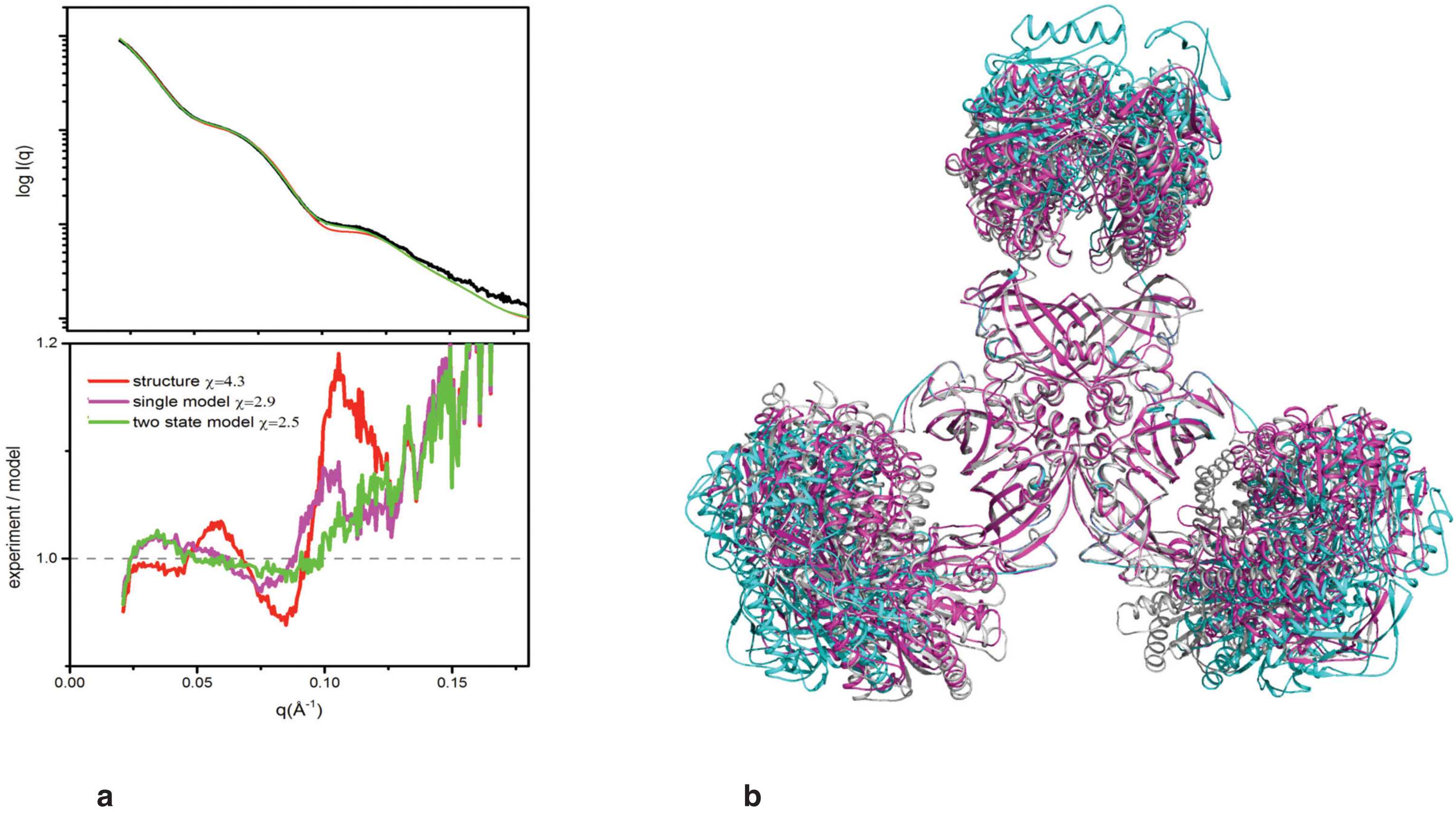

Supplementary Figure 9: Small-angle X-ray scattering analysis of PaaZ

a The cryoEM derived model of PaaZ fits the experimental SAXS profile with a chi value of 4.3. A single state model obtained from BILBOMD fits with a chi value of 2.9, whereas as two state model (A and B) fits slightly better with a chi value of 2.5.

b Superposition of cryoEM derived model (shown in grey) and model A (shown in magenta) and Model B (shown in cyan) obtained from BILBOMD.

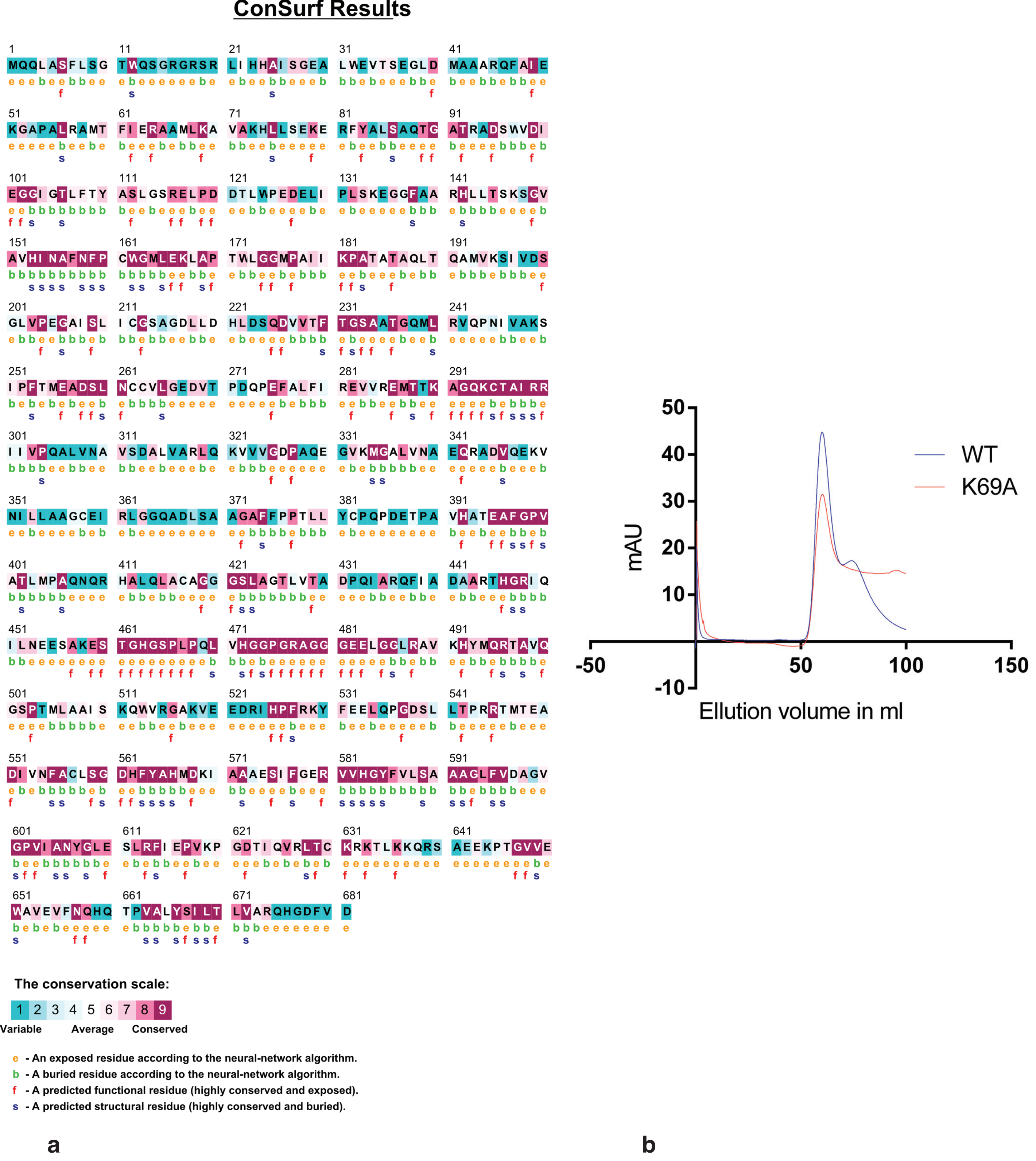

Supplementary Figure 10

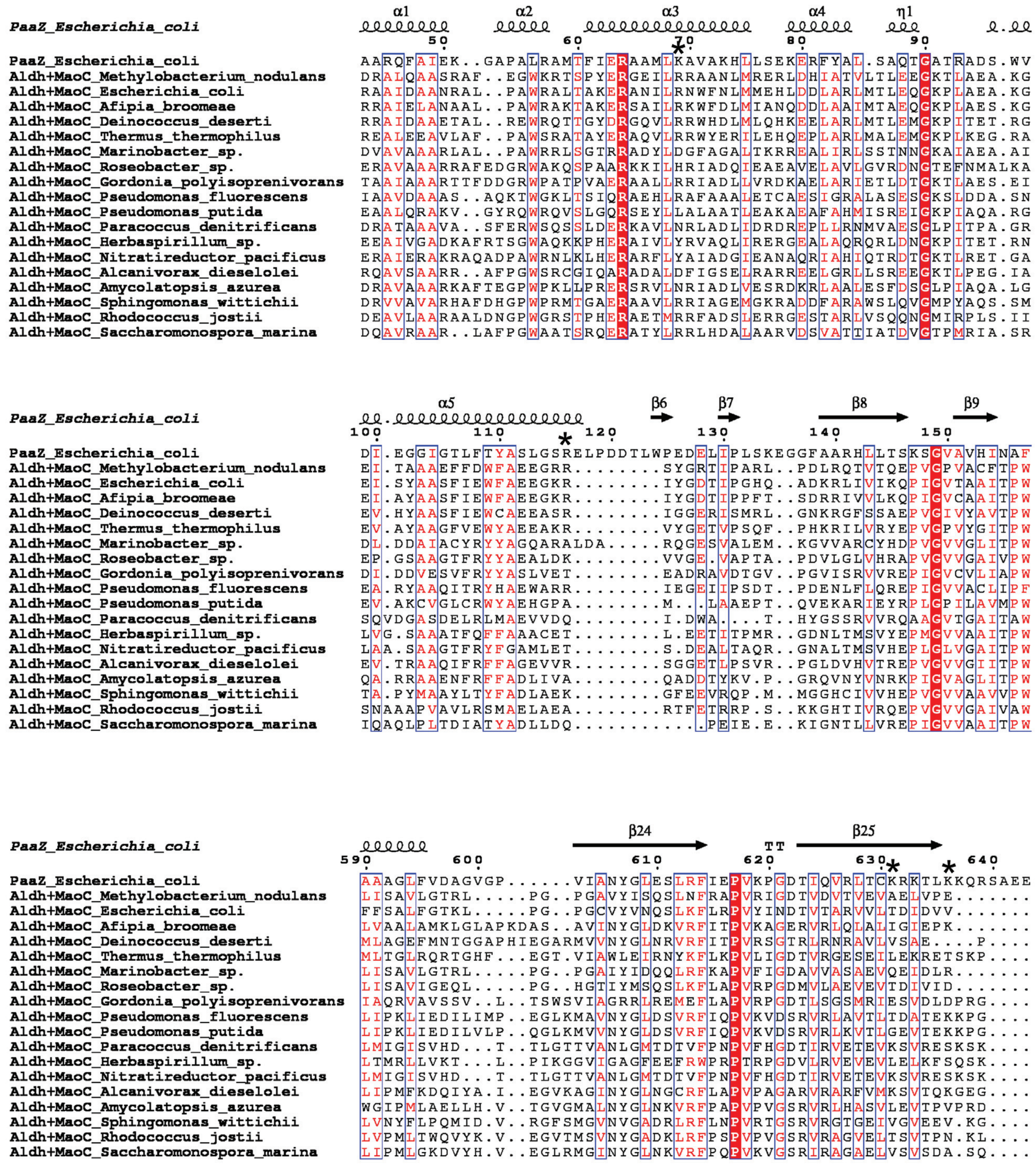

c

Supplementary Figure 10: Sequence analysis and gel filtration profile of PaaZ

a Evolutionary conservation of PaaZ homologues as analysed by Consurf

b Gel filtration profiles of PaaZ WT and K69A mutant show similar elution profiles.

c Sequence analysis of individual dehydrogenase and hydratase domains against PaaZ.

Alignment of 20 representative homologues of PaaZ containing individual gene products of aldehyde dehydrogenase, enoyl-CoA hydratase domains. K69, R116, K631 and K636 are conserved only in fused PaaZ and its homologues while these amino acids are seen not to be under the evolutionary pressure in individual gene products of aldehyde dehydrogenase, enoyl-CoA hydratase domains.
